# High-fat feeding rather than obesity drives taxonomical and functional changes in the gut microbiota in mice

**DOI:** 10.1186/s40168-017-0258-6

**Published:** 2017-04-08

**Authors:** Liang Xiao, Si Brask Sonne, Qiang Feng, Ning Chen, Zhongkui Xia, Xiaoping Li, Zhiwei Fang, Dongya Zhang, Even Fjære, Lisa Kolden Midtbø, Muriel Derrien, Floor Hugenholtz, Longqing Tang, Junhua Li, Jianfeng Zhang, Chuan Liu, Qin Hao, Ulla Birgitte Vogel, Alicja Mortensen, Michiel Kleerebezem, Tine Rask Licht, Huanming Yang, Jian Wang, Yingrui Li, Manimozhiyan Arumugam, Jun Wang, Lise Madsen, Karsten Kristiansen

**Affiliations:** 1 BGI-Shenzhen, Shenzhen, 518083 China; 2 Laboratory of Genomics and Molecular Biomedicine, Department of Biology, University of Copenhagen, DK-2100 Copenhagen, Denmark; 3 National Institute of Nutrition and Seafood Research (NIFES), Postboks 2029, Nordnes, N-5817 Bergen, Norway; 4 Laboratory of Microbiology, Wageningen University, 6701 AK Wageningen, The Netherlands; 5 National Research Centre for the Working Environment, DK-2100 Copenhagen, Denmark; 6 National Food Institute, Technical University of Denmark, DK-2800 Kongens Lyngby, Denmark; 7 The Novo Nordisk Foundation Center for Basic Metabolic Research, University of Copenhagen, Copenhagen, DK-2100 Denmark; 8 James D. Watson Institute of Genome Sciences, Hangzhou, 310058 China; 9 Present address: Danone Research, TI Food and Nutrition, Paris, France

**Keywords:** C57BL/6J mice, 129S6/Sv mice, Obesity, High-fat feeding, Microbiota, Microbiome, Indomethacin

## Abstract

**Background:**

It is well known that the microbiota of high-fat (HF) diet-induced obese mice differs from that of lean mice, but to what extent, this difference reflects the obese state or the diet is unclear. To dissociate changes in the gut microbiota associated with high HF feeding from those associated with obesity, we took advantage of the different susceptibility of C57BL/6JBomTac (BL6) and 129S6/SvEvTac (Sv129) mice to diet-induced obesity and of their different responses to inhibition of cyclooxygenase (COX) activity, where inhibition of COX activity in BL6 mice prevents HF diet-induced obesity, but in Sv129 mice accentuates obesity.

**Results:**

Using HiSeq-based whole genome sequencing, we identified taxonomic and functional differences in the gut microbiota of the two mouse strains fed regular low-fat or HF diets with or without supplementation with the COX-inhibitor, indomethacin. HF feeding rather than obesity development led to distinct changes in the gut microbiota. We observed a robust increase in alpha diversity, gene count, abundance of genera known to be butyrate producers, and abundance of genes involved in butyrate production in Sv129 mice compared to BL6 mice fed either a LF or a HF diet. Conversely, the abundance of genes involved in propionate metabolism, associated with increased energy harvest, was higher in BL6 mice than Sv129 mice.

**Conclusions:**

The changes in the composition of the gut microbiota were predominantly driven by high-fat feeding rather than reflecting the obese state of the mice. Differences in the abundance of butyrate and propionate producing bacteria in the gut may at least in part contribute to the observed differences in obesity propensity in Sv129 and BL6 mice.

**Electronic supplementary material:**

The online version of this article (doi:10.1186/s40168-017-0258-6) contains supplementary material, which is available to authorized users.

## Background

Evidence has accumulated that the gut microbiota is an important environmental factor contributing to obesity by altering host energy harvest and storage [1–4], and transplantation experiments where transfer of “obese microbiota” induces fat mass accumulation in recipient mice have underscored the importance of microbiota composition in obesity development [1, 3, 5–9]. High-fat (HF) feeding and obesity were initially reported to be associated with a lowered ratio of *Bacteroidetes* to *Firmicutes*, but this view has been challenged by subsequent studies [2, 10]. Recent studies have further indicated that obesity is correlated with decreased microbial diversity or richness [11–13]. The composition of the gut microbiota may, in part, be modulated by the genetic background [14–16] also illustrated by the alterations in the gut microbiota profiles observed in TLR5 knockout mice [7], TLR2 knockout mice [5], NOD mice [17], *ob/ob* mice [3], and *db/db* mice [18, 19] compared with wild-type mice. However, in these mouse models, altered feed intake may also contribute to the observed changes in the gut microbiota. Yet, in some cases, transplantation of the microbiota from the knockout mice into wild-type recipient mice conferred a phenotype similar to that of the donor [7]. Still, long-term habitual dietary intake is thought to be one of the strongest drivers of the gut microbial composition in humans [20] as well as mice [21], and especially intake of fat is a strong driver of changes in the gut microbiota [15]. However, as the amount of fat also determines obesity development, the commonly used models of HF diet-induced obesity cannot distinguish whether changes in the microbiota result from the obese state or from HF feeding.

The obesity-prone C57BL/6JBomTac (BL6) mouse strain and the obesity-resistant mouse strain 129S6/SvEvTac (Sv129) are among some of the most commonly used strains for studies on genetic and diet-induced obesity. We have shown that inhibition of cyclooxygenase activity accentuated HF feeding-induced obesity in the obesity-resistant Sv129 mice by reducing diet-induced thermogenesis and induction of UCP1 expression in inguinal white adipose tissue [22], whereas inhibition of cyclooxygenase activity in the normally obesity-prone BL6 mice prevented HF feeding-induced obesity [23].

To gain further insight into diet- and obesity-associated changes in the gut microbiota, and to distinguish whether the observed changes resulted from the obese state or the HF feeding*,* we took advantages of the different susceptibility of these two mouse strains to diet-induced obesity and of their different responses to inhibition of cyclooxygenase activity. Our results indicate that changes in gut microbiota largely reflect HF feeding and not obesity.

## Results

### Experimental setup and construction of a gut metagenome reference set

**Fig. 1:**
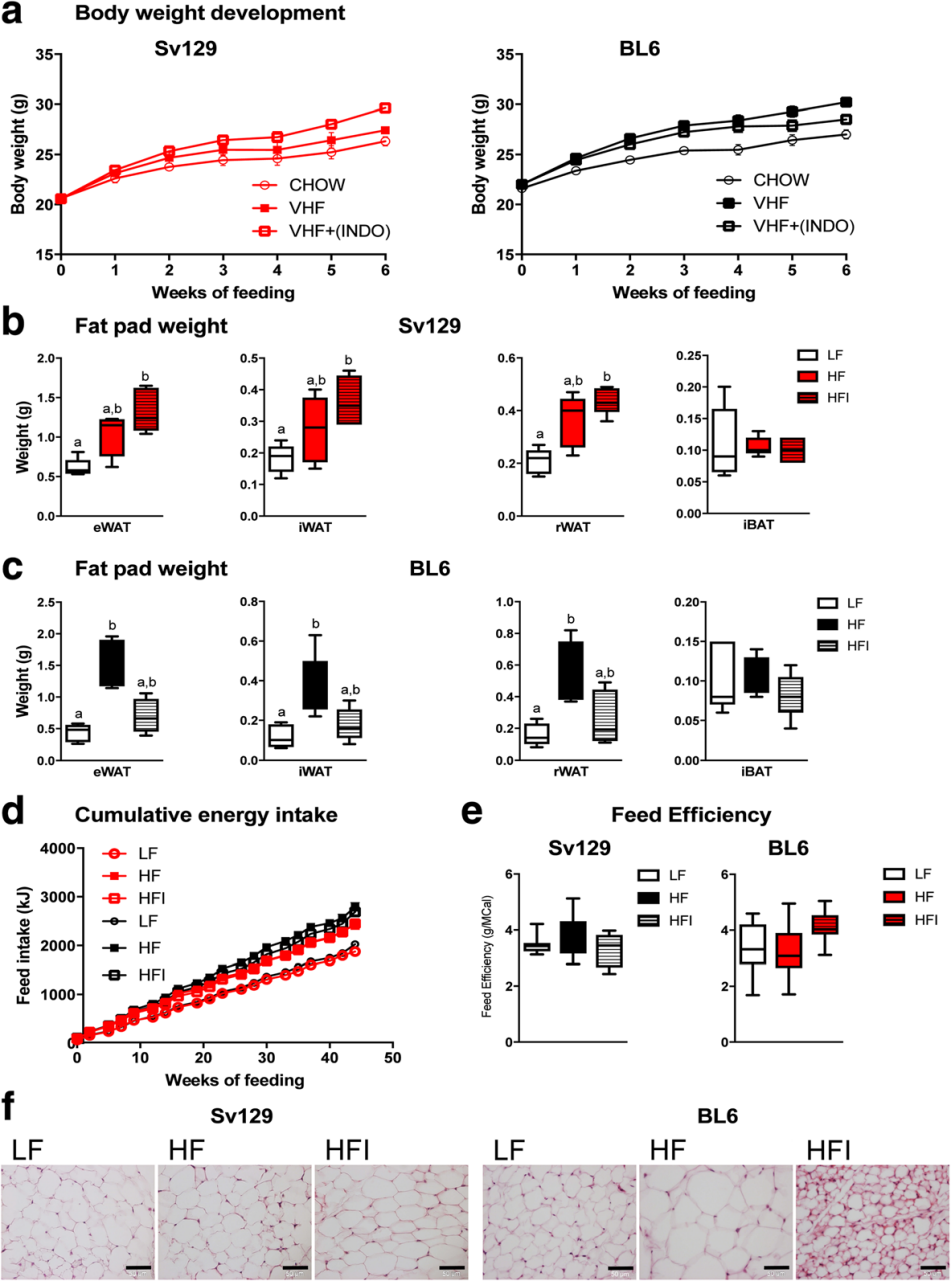
Body weight gain, adipose tissue weight, and adipose tissue histology. **a** Body weight gain of Sv129 and BL mice fed low-fat (LF), high-fat (HF), or HF + indomethacin (HFI) diets. **b**, **c** Tissue weights of epididymal (eWAT), inguinal (iWAT), retroperitoneal (rWAT), and interscapular brown (iBAT) adipose depots in **b** Sv129 and **c** BL mice fed low-fat (LF), high-fat (HF), or HF + indomethacin (HFI) diets. *Error bars* represent s.e.m.. *Shared letters on the bars* indicate *P* ≥ 0.05, whereas *different letters* denote significant differences between the groups (<0.05), Kruskal-Wallis with Dunns post hoc test. **d** Cumulative feed intake. **e** Energy efficiency. **f** Hematoxylin/eosin staining of iWAT tissues from Sv129 and BL mice fed LF, HF or HFI diets. *Scale bar* = 50 μm

To distinguish changes in the gut microbiota that occur in response to an obesogenic diet from changes due to obesity development, we exploited the different propensity of BL6 and Sv129 mice to develop diet-induced obesity and their divergent responses to treatment with the general cyclooxygenase inhibitor indomethacin. The mice were maintained on a low-fat (LF) diet or fed a HF diet supplemented (HFI) or not (HF) with indomethacin. In agreement with earlier findings [22], inclusion of indomethacin accentuated the high-fat diet-induced increase in weight, white adipose tissue (WAT) mass, and hypertrophy in Sv129 mice (Fig. 1). By contrast, indomethacin supplementation prevented high-fat diet-induced increase in weight, WAT mass, and hypertrophy in BL6 mice (Fig. 1) [23].

Bacterial DNA was isolated from fecal samples from 54 mice from the different groups (30 Sv129 mice: 10 fed the LF diet, 10 fed the HF diet, and 10 fed the HFI diet ; 24 BL6 mice: 7 fed the LF diet, 8 fed the HF diet, and 9 fed the HFI diet) and subjected to whole genome sequencing (WGS) using the Illumina HiSeq2000 platform [24]. In total, 200.91 Gb high-quality data were generated with an average of 3.72 Gb (46.10 million reads) per sample. We employed de novo assembly as previously described [15] to generate a non-redundant gene catalog containing 793,847 genes (Additional file 1: Table S1). A rarefaction analysis revealed a curve approaching saturation with 45 samples, and ICE and Chao 1 indices indicated that we captured 99.53% of the total gut microbial genes in the cohort (Additional file 2: Figure S1). We carried out taxonomical assignment and functional annotation using the Integrated Microbial Genomes (IMG) database (v3.4) [25], the NR database (v3), and KEGG [26] database (release 59.0), respectively. 6.53% of the genes in the catalog, which covered 14.85% in gene abundance, could be annotated using the IMG database, while 75.14% of the genes could be annotated using the NR database. At the functional level, we identified 4846 KEGG orthologues (KOs) covering 46.43% of genes. For each sample, an average of 46.9 ± 0.2% (mean ± s.e.m.) of genes could be annotated in the KEGG database. The taxonomic and functional profiles were constructed by summarizing the relative abundance of genes.

### Diet-induced and strain-dependent changes in the gut microbiota

**Fig. 2:**
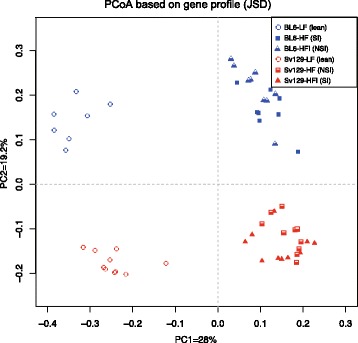
PCoA analysis of all the samples based on gene profiles. The *different colors* and *shapes* designate samples from different subgroups, while the *empty*, *half-filled*, and *full-filled points* correspond to mice characterized as “lean,” or with “no significant increase in adipose tissue mass (NSI),” and “significant increase adipose tissue mass (SI).” The PCoA demonstrates how mouse strain and diet are main drivers for separation at the gene level

Gene, genus, and KO profile-based principal coordinates analysis (PCoA) (Fig. 2 and Additional file 3: Figure S2) pointed to dietary fat content as the principal separating factor (PC1) and strain-specific differences between Sv129 and BL6 mice as the secondary separating factor (PC2). The PCoA further demonstrated that there was no significant separation between the HF- and the HFI-fed mice based on the microbiome gene profiles (Fig. 2) and by Wilcoxon rank-sum testing (Additional file 4: Figure S3 and Additional file 5: Figure S4), despite a clear effect of indomethacin on obesity development.

A PERMANOVA test similarly demonstrated that diet had a larger impact on the gut microbiota than strain and importantly that indomethacin supplementation did not significantly affect the gut microbiota composition at the gene level, the genus level, and the KEGG level (Additional file 6: Table S2).

**Fig. 3:**
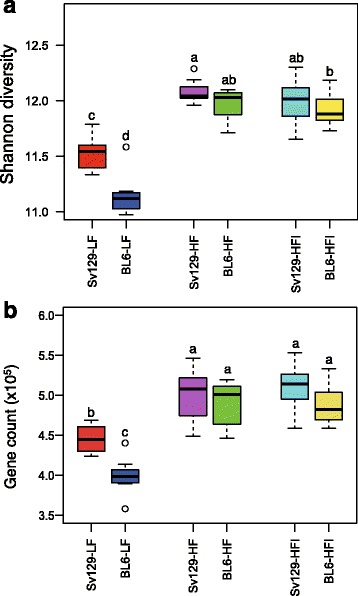
**a** Alpha diversity and **b** Gene count in relation to mouse strain and diet. Alpha diversity was calculated based on the gene profiles using the Shannon index. In mice fed a low-fat (LF) diet, Sv129 mice exhibited a significantly higher alpha diversity than BL6 mice. High-fat (HF) diet increased alpha diversity of the gut microbiome significantly in both strains of mice, so that no significant differences in alpha diversity were observed after HF feeding. Supplementation with indomethacin did not lead to significant changes of alpha diversity. Gene count in LF-fed Sv129 mice was significantly higher than in BL6 mice. HF feeding led to a significant increase in gene count in both mouse strains and eliminated the difference in gene count observed in LF mice. Statistical differences were analyzed by unpaired Wilcoxon rank-sum test (with FDR correction). Statistically significant differences (*P* < 0.05) between groups are denoted with different letters (*a*, *b*, *c*, *d*) on the top of the graphic boxes. *Boxes* denote the interquartile range (IQR) between the first and third quartiles (25th and 75th percentiles, respectively) and the *line inside* denotes the median

Comparison of the gut microbiomes of mice fed a LF diet revealed significantly higher alpha diversity of the samples from Sv129 mice than BL6 mice (Fig. 3a). Feeding the mice a HF diet led to a significantly increased alpha diversity at both the gene and KO levels in both strains with a relatively larger increase in BL6 mice. As the increased alpha diversity was more pronounced in BL6 mice, the difference in alpha diversity became non-significant in HF-fed mice. Addition of indomethacin to the HF diet did not significantly influence alpha diversity (Fig. 3a and Additional file 7: Figure S5). We further investigated differences and similarity of the gene distribution in the two mouse strains and how the HF diet impacted on the composition of the gut microbiota. Wilcoxon testing demonstrated that a large fraction of the microbial genes (444,171; 60.18%) was found in similar relative abundance in both strains fed the LF diet. 202,729 (27.47%) of the genes had higher relative abundance in LF-fed Sv129 mice than in LF-fed BL6 mice, whereas 91,223 (12.36%) of the genes were present in higher abundance in LF-fed BL6 mice. Of the genes that were shared between the two mouse strain fed the LF diet, 320,199 (72.09%) maintained the same relative abundance in the two strains in response to HF feeding, whereas 58,027 (13.06%) increased in the Sv129 mice and 55,546 (12.51%) increased in the BL6 mice. 10,399 (2.34%) became undetectable after HF feeding. Of the 202,729 genes that were enriched in the LF-fed Sv129 mice, 122,577 (27.60%) were found in equal relative abundance in Sv129 and BL6 mice after HF feeding, whereas 2866 (0.65%) became undetectable. Interestingly, a small subset of the genes 4693, 1.06%) enriched in Sv129 mice on a LF diet became more abundant in the BL6 mice. Finally, of the 91,223 genes selectively enriched in the BL6 mice fed a LF diet, 41,458 (9.33%) were found I equal relative abundance after HF feeding in both strain, 45,087 (10.15%) remained more abundant in the BL6 mice, whereas 3463 (0.78%) became more abundant in the Sv129 mice. 1215 (0.27%) of the genes enriched in BL6 mice were undetectable after HF feeding (Additional file 8: Figure S6).

Obesity has been reported to be associated with a decreased microbial gene count [11, 12]. In LF-fed mice, the gene count was significantly higher in the Sv129 mice than in the BL6 mice (Fig. 3b). HF feeding with or without indomethacin supplementation increased the gene count in both strains. Thus, an increase in gene count was observed in response to HF feeding irrespective of whether the mice remained lean or became obese. Of note, after HF feeding, no significant difference in gene count between the two strains was observed (Fig. 3b).

Consumption of HF diets induced taxonomical changes independently of strain and obesity development. The Wilcoxon rank-sum test (*P* < 0.05) demonstrated that the HF diet induced the same directional changes in relative abundances of 89 out of 128 genera (69.5%) in both mouse strains, whereas only 8 (6.3%) exhibited changes exclusively in BL6 mice, and 11 (8.6%) exhibited changes exclusively in Sv129 mice. The remaining identified 20 genera (15%) were unchanged.

In keeping with previous observations [1–4, 27], the five most abundant phyla in both Sv129 and BL6 mice included *Bacteroidetes, Firmicutes, Verrucomicrobiota, Proteobacteria*, and *Actinobacteria*, and HF feeding led to an increased *Firmicutes*:*Bacteroidetes* ratio (Additional file 9: Figure S7). In mice fed a standard LF diet, *Firmicutes* were present at a higher abundance in Sv129 than in BL6 mice, but the abundance increased to similar levels after HF feeding. Further, *Verrucomicrobia* were more abundant in BL6 mice than in Sv129 mice, but no alterations in abundance were observed in response to HF feeding (Additional file 9: Figure S7). Of the less abundant phyla, we observed a significant increase in relative abundance of *Spirochaetes, Fusobacteria, Synergistetes,* and *Euryarchaeota* in response to HF feeding in both strains (Additional file 10: Figure S8).

In agreement with previous studies [15], the five most abundant genera in BL6 and Sv129 included *Bacteroides*, *Clostridium*, *Akkermansia*, *Roseburia*, and *Lactobacillus* (Additional file 5: Figure S4). In both BL6 and Sv129 mice, HF feeding decreased the relative abundance of *Bacteroides* but increased the relative abundance of *Clostridium, Roseburia*, and *Lactobacillus* (Additional file 5: Figure S4). Of the less abundant genera, we noted dramatic decrease in the relative abundance of *Tannerella, Prevotella*, and *Parabacteroides* in both strains in response to HF feeding (Additional file 11: Figure S9). By contrast, the relative abundance of *Oscillibacter, Eubacterium, Ruminococcus, Pseudoflavonifractor, Blautia, Dorea, Anaerotruncus, Subdoligranulum*, and *Faecalibacterium* increased in response to HF feeding (Additional file 11: Figure S9).

The most abundant identified species comprised *Akkermansia muciniphila*, *Lachnospiraceae bacterium* 3_57fAA_CT1, *Tannerella* sp. 6_1_58FAA_CT1, *Ruminococcaceae bacterium* D16, and *Oscillibacter valericigenes* (Additional file 12: Figure S10). Changes in less abundant identified species are shown in Additional file 13: Figure S11. Of note, the relative abundance of *A. muciniphila,* reported to maintain gut barrier function and associated with resistance to diet-induced obesity [28], was surprisingly lower in Sv129 than in BL6 mice on both LF and HF diets (Additional file 12: Figure S10). Presently, it is difficult to reconcile this finding with the consistent observations that *A. muciniphila* seems to protect against low-grade inflammation and diet-induced obesity, but the increased abundance of *A. muciniphila* may reflect a homeostatic response to counteract low-grade inflammation in the obesity-prone BL6 mice. We observed a striking decrease in the relative abundance of *Tannerella* sp. 6_1_58FAA_CT1 in response to HF diets in both Sv129 and BL6 mice (Additional file 12: Figure S10).

### Genera selectively enriched in the Sv129 and BL6 mice and diet-induced functional changes of the microbiota

**Fig. 4:**
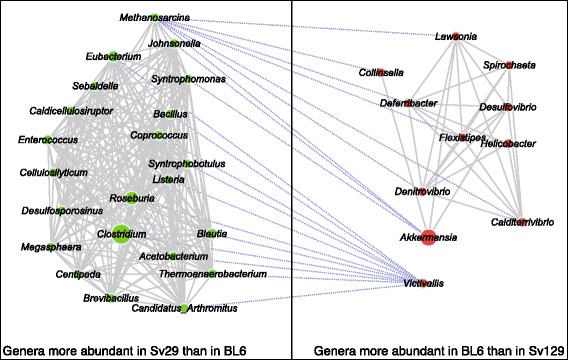
Network of the genera characterizing Sv129 and BL6 mice. The *green circles* represent genera present in higher abundance in Sv129 mice than in BL6 and *red circles* genera present in higher abundance in BL6 mice than in Sv129. The *area of the circle* represents the relative abundance of the genus. The *solid line* represents a positive correlation between two genera, while a *dashed line* represents a negative correlation

To further characterize the microbiota of the two mouse strains, we identified genera with higher abundance in each of the strains, and co-occurrence networks in each strain fed the LF diet (*P* < 0.05) (Fig. 4). *Clostridium* and *Roseburia* were found enriched in Sv129 mice, and these two genera are reported as butyrate-producing bacteria [29] suggesting that the potential for butyrate formation was selectively enriched in Sv129 mice. The butyryl-CoA transferase (BCT), catalyzing the last step in the pathway leading to butyrate formation, can be used as a gene marker for butyrate production [30]. To further examine butyrate formation potential, we examined the relative abundance of the gene encoding the BCT enzyme [31] in Sv129 and BL6 mice.

**Fig. 5:**
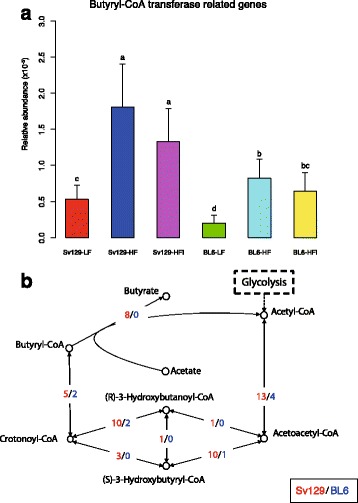
Relative abundance of butyryl-CoA transferase related genes in Sv129 and BL6 mice and pathways catalyzing conversion of acetyl-CoA to butyrate. **a** The relative abundance of butyryl-CoA transferase genes was higher in Sv129 than BL6 mice. Of note, HF feeding increased the relative abundance of butyryl-CoA transferase genes in both strains. Statistical differences were analyzed by unpaired Wilcoxon rank-sum test (with FDR correction). Statistically significant differences (*P* < 0.05) between groups are denoted with different letters (*a*, *b*, *c*, *d*) on the top of the graphic boxes. **b** The pathways catalyzing conversion of acetyl-CoA to butyrate comprise eight enzymatic reactions, and the numbers of the enzymes catalyzing these reactions in Sv129 and BL6 mice are marked in *different colors*, Sv129 in *blue*, and BL6 in *red*. The number of the enzymes included in this pathway module is markedly higher in Sv129 mice than in BL6 mice

In our gene catalog, we identified 12 genes matching the BCT sequence using BLAST using thresholds of 70% identity and 70% coverage at the amino acid sequence level. The relative abundance of these 12 genes was summarized as a proxy representing butyrate formation capacity (Fig. 5a), which indicated that the abundance of BCT was significantly higher in Sv129 mice than in BL6 mice, but in both stains, the abundance of BCT unexpectedly increased when the mice were fed HF diets. To further analyze the potential capacity for butyrate production, we defined a butyrate formation module including enzymes catalyzing 8 reactions from acetyl-CoA to butyrate (Fig. 5b). The relative abundance of the 11 KOs comprising this module was calculated according to the different subgroups. In line with the results shown in Fig. 5b, the relative abundance of KOs comprising this butyrate formation module was higher in Sv129 mice than in BL6 mice (Additional file 14: Figure S12). This suggests that differences in butyrate production of the gut microbiota in Sv129 and BL6 mice might contribute to the different phenotypes in relation to obesity propensity. We used a previously reported *z* score method [32] to further analyze diet-dependent differences between the two strains at the functional level. Main differences are listed in Additional file 15: Table S3. These analyses indicated that the metabolism of short chain fatty acids (SCFAs) was differently affected by the HF diet in the two strains. In keeping with results on the potential for butyrate production, HF feeding was associated with a general increase in the abundance of genes involved in butyrate metabolism in both strains (Additional file 14: Figure S12), whereas the abundance of genes involved in propionate metabolism module, associated with increased energy harvest, increased in BL6 mice in response to HF feeding (Additional file 15: Table S3), possibly at least in part contributing to the sensitivity of the BL6 strain to HF diets. Still, in relation to obesity propensity, more experiments are clearly needed to explain the increase in the abundance of BCT in response to HF feeding.

KEGG level 1 pathway analyses revealed additional HF diet-induced changes, independent of strain and obesity. The relative abundance of KOs associated with cellular processes and environmental information processes increased after HF diet feeding, while the abundance of KO associated with general metabolism declined (Additional file 4: Figure S3). To further analyze the affected metabolic pathways, we used *z* score method [32] for functional analysis at the level of pathways and modules followed by detailed analysis of KO changes in each selected pathway and module. Independent of mouse strain, HF feeding increased the abundance of genes involved in pathways and modules related to fatty acid metabolism, cell mobility, transport, methane metabolism, and xenobiotic degradation, as well as decreases in the abundance of genes involved in translation and vitamin biosynthesis (Additional file 16: Table S4 and Additional file 17: Table S5).

Utilization of the glycerol moiety of the triglycerides is required to extract energy from triglycerides in HF diets in an anaerobic environment. To investigate if the gut microbiota adapts to increased glycerol utilization in response to a HF diet, we used KEGG and NCBI to search for the key enzyme in glycerol utilization, glycerol kinase, in the most abundant genera. Of note, glycerol kinase was found in 18 of the 25 most abundant genera (Additional file 18: Table S6). Moreover, the majority of genera possessing genes encoding glycerol kinase was enriched in mice fed HF diets, whereas genera unable to utilize glycerol were enriched in low-fat diet-fed mice (Additional file 18: Table S6). Together, this suggests that increased capacity for glycerol utilization characterizes HF diet-induced changes in the gut microbiota in both mouse strains.

Bile acids play an important role in fat metabolism in the intestine, and HF feeding may lead to an increased requirement for bile acid 7α-dehydroxylation. Accordingly, reads classified as *Clostridium scindens*, *Clostridium hiranonis*, *Clostridium hylemonae*, and *Clostridium leptum*, all reported to accelerate bile acid 7α-dehydroxylation [33, 34], were found in higher abundances after HF feeding (Additional file 19: Figure S13). Furthermore, the relative abundance of the *Clostridium* genus was increased in both mouse strains in response to HF diet (Additional file 5: Figure S4).

## Discussion

Human cross-sectional studies and animal studies have demonstrated that the gut microbiota of obese individuals differs from that of lean individuals and also show that diet strongly influences the composition of the gut microbiota. Still, these studies do not allow to clearly determine whether the different composition of the gut microbiota is a cause or a consequence of the obese state even though transplantation experiments have demonstrated that an “obese” microbiota may precipitate obesity in the recipient. In this study, the divergent effect of HF diets with and without indomethacin in BL6 and Sv129 mice allowed us to separate alterations in the gut microbiota induced by the change from a LF diet to a HF diet from alterations elicited by obesity showing that the changes in gut microbiota reflected an effect of HF feeding and not of obesity. This finding is in agreement with an earlier study using 16S rRNA gene amplicon sequencing to compare the effect of HF feeding on RELM knockout and wild-type mice on a mixed 129Svev/C57BL/6 background, namely that HF feeding and not obesity is the driver of the changes in the composition of the gut microbiota [35]. A similar conclusion was reached by Clavel et al. based on fluorescence in situ hybridization [36]. Our study adds to these finding by providing more detailed information at the gene level using metagenomic whole genome sequencing.

In rats, it has been reported that treatment with indomethacin was associated with changes in the gut microbiota, including gain of *Enterococcus faecalis*-related bacteria and loss of segmented filamentous bacteria [37]. However, our results (Additional file 20: Figure S14) revealed no significant changes in the composition of *E. faecalis*-related bacteria in mice fed a HF diet supplemented with indomethacin. This difference between mice and rats may in part be related to the absence of a gall bladder in rats, but obviously, other species-related differences may play a role. A recent study reported on the acute and chronic effects of indomethacin supplementation in BL6 mice based on 16S ribosomal RNA (rRNA) gene amplicon sequencing [38]. Using an acute dose about 10 times higher than the dosage used in the present experiments, the authors found that the abundance of *Peptococcaceae* in the fecal samples increased already after 6 h. Exposure for 7 days using a dosage similar to the one used here resulted in no significant changes in the fecal microbiome, whereas an increased abundance of *Peptococcaceas* was observed in samples from cecum and the large intestine [38]. This finding and the clear effects of the microbiota on the metabolism of indomethacin suggest that some of the strain-specific effects of indomethacin supplementation may relate to differences in composition of the microbiota and changes in the gut not reflected in fecal samples.

Comparison between the microbiota of the obesity-prone BL6 and the obesity-resistant Sv129 mice revealed that Sv129 mice had higher gene count than BL6 mice (Fig. 3b). Low gene count has been associated with a more obese and inflamed phenotype in mice and humans [12, 39]. Furthermore, a decline in the abundance of genes involved in butyrate production has been noted as a common feature of low gene count obese individuals [12] as well as in type 2 diabetic Han Chinese [40], and a generally reduced potential for butyrate production in the gut microbiota has been associated with a number of human diseases [41]. Interestingly, dietary intake of high amounts of butyrate has been reported to improve insulin sensitivity and counteract HF diet-induced obesity [42, 43], but whether gut microbial synthesis can raise butyrate levels in circulation to the same degree remains questionable. To add to the complexity of the effects of butyrate on obesity development, butyrate production was initially associated with increased energy harvest from the gut and obesity [3], and increased production of butyrate has been reported in obese individual [10]. Yet, analysis of the potential for butyrate production in the gut microbiota of Sv129 and BL6 mice demonstrated that the obesity-resistant Sv129 mice harbored a gut microbiota with a potentially higher capacity for butyrate production than the obesity-prone BL6 mice. Thus, the relative abundance of the genes encoding the enzyme catalyzing the last step leading to butyrate formation, BCT, was significantly higher in Sv129 mice than in BL6 mice. Furthermore, by defining a butyrate formation module including enzymes catalyzing eight reactions from acetyl-CoA to butyrate, we demonstrated that the relative abundance of the genes comprising this module was higher in Sv129 mice than BL6 mice. This suggests that differences in butyrate production in the gut of Sv129 and BL6 mice may contribute to the different phenotypes in relation to obesity propensity. However, our finding that the abundance of BCT increased in response to HF feeding clearly indicates that more experiments are needed to provide a comprehensive understanding of the role of butyrate production in obesity development.

In mice fed a LF diet, we noted characteristic differences between Sv129 and BL6 mice. Whereas *Firmicutes* were present at a higher abundance in Sv129 than in BL6 mice, *Verrucomicrobia* were more abundant in BL6 mice than in Sv129 mice. Surprisingly, we observed that the relative abundance of *A. muciniphila*, reported to maintain gut barrier function and associated with resistance to diet-induced obesity [28], was lower in the obesity-resistant Sv129 than in BL6 mice on both LF and HF diets. This finding underscores that even though colonization by this bacterium is reported to counteract diet-induced obesity [28, 44, 45], the function of this bacterium may well depend on a specific community environment, as also indicated by the large differences in the relative abundance of *A. muciniphila* in fecal samples from mice kept in different housing facilities [15].

Extraction of energy from triglycerides in an anaerobic environment depends on the metabolism of glycerol. Using KEGG and NCBI to search for key enzymes in glycerol utilization in the most abundant genera, we found that the genes encoding glycerol kinases, acylglycerol kinase, or diacylglycerol kinase were found in 10 of the 25 most abundant genera, and 8 of these genera were enriched in mice fed HF diets whereas genera unable to utilize glycerol were enriched in mice fed the low-fat diet. This suggests that such adaptation contributes to the strain-independent HF diet-induced changes in the gut microbiota. Similarly, HF feeding led to an increase in the abundance of *Clostridium* species reported to accelerate bile acid 7α-dehydroxylation [33, 34], may be associated with a need for bile acid metabolism in response to the intake of a diet high in triglycerides, but more work is clearly needed to establish whether HF feeding alter bile acid metabolism.

## Conclusions

This study demonstrates that ingestion of a HF diet is a major driver of changes in the gut microbiota in mice, irrespective of whether or not the mice develop obesity. Of note, the gene count in LF diet-fed Sv129 mice was higher than that of BL6 mice. Compared with BL6 mice, the microbiome of Sv129 mice have a higher abundance of genes involved in butyrate production. Butyrate plays a key role in gut health and low gene count obese individuals, and type 2 diabetic patients seem to have less potential for butyrate production than high gene count [12, 40]. Thus, our findings suggest that differences in the capacity for butyrate production between Sv129 mice than in BL6 may contribute to the different propensity of these two mouse strains to develop diet-induced obesity. However, further analyses of the actual production of butyrate in the gut and levels of butyrate in circulation are needed to corroborate this notion. In a broader sense, our findings open the possibility that changes in caloric intake/fat intake associated with both diet-induced and genetically dependent obesity may be the driver for the observed changes in the gut microbiota, and that such diet-dependent changes subsequently in transplantation experiments may confer the obese phenotype. While differences in the gut microbiota may contribute to the different propensity for obesity development in the two mouse strains, differences in the genetic make-up of these two strains may also play a role, directly or indirectly via effects on the composition of the gut microbiota.

## Methods

### Test compounds and diets

Low-fat (Ssniff EF R/M control) and high-fat (Ssniff EF R/M acc D12492) diets were obtained from Ssniff Spezialdiäten GmbH (Germany). The low-fat (LF) diet contained 70 energy (e)% carbohydrates, 20 e% protein, and 10 e% fat, and the high-fat (HF) diet contained 21 e% carbohydrates, 19 e% protein, and 60 e% fat. For the HF diet supplemented with indomethacin (HFI), 0.0016 g/100 g was included in the diet.

### Animals and housing

Twenty-four male C57BL/6JBomTac and 30 male 129S6/SvEvTac mice, 10 weeks old, were obtained from Taconic (Ry, Denmark). All mice were individually caged with sawdust bedding, kept under controlled environmental conditions (temperature 26 ± 0.5 °C, 12/12-h light/dark cycle), and had free access to feed and water.

After 1 week of acclimatization, mice of each strain were placed in three groups (10 LF-, 10 HF-, and 10 HFI-fed Sv129 mice; 7 LF-, 8 HF- and 9 HFI-fed BL6 mice) and fed these different diets for 6 consecutive weeks. Body weight was recorded twice a week, and feed intake was recorded weekly. All mice were observed at least twice a day for any abnormalities in clinical appearance. At the end of the experiment, mice were anesthetized with isoflurane (Isoba-vet, Schering-Plough, Denmark) and euthanized by cardiac puncture. Relevant adipose tissues were immediately dissected, weighed, flash-frozen in liquid nitrogen, and stored at −80 °C. The animal experiment was approved by the National State Board of Biological Experiments with Living Animals (Norway and Denmark) and carried out in accordance with the approved guidelines.

### DNA extraction

Fresh feces was sampled before the termination and immediately frozen at −80 °C. DNA extractions were performed on 200 mg of feces per sample using the Macherey-Nagel Nucleospin Soil kit. Extractions were carried out according to the kit protocol except that cell lysis was done by bead beating the samples two times for 30 s with incubation for 2 min on ice between the bead beatings. The resulting concentrations of genomic DNA were measured by nanodrop, and DNA integrity examined by agarose gel electrophoresis. Metagenomic sequencing was conducted using HiSeq 2000 and 90-bp PE strategy [24].

### De novo assembly and gene prediction

After removing adapters, low-quality reads and reads that belong to the host were removed, 46,100,196 ± 977,672 (mean ± s.e.m.) high-quality reads were obtained. These high-quality reads from the 54 samples were then assembled to contigs using SOAPdenovo (v1.06) by employing the same parameters that were used in MetaHIT gene catalog [24]. For each sample, 53,226 ± 2,097 (mean ± s.e.m.) contigs were obtained with a length 102,946,516 ± 2,678,730 bp (mean ± s.e.m.). GeneMark (v2.7) was employed to predict open reading frames (ORFs).

### Construction of a gut metagenome reference

To explore the metagenomic information of the mouse gut microbiota, we first generated a metagenomic catalog based on the samples obtained in the present study. All ORFs predicted from the 54 samples were merged and aligned to each other using BLAT. Gene pairs with greater than 95% identity (no gap allowed) and aligned reads covering over 90% of the shorter reads were grouped together. The longest ORF in each group was used to represent the group, and the other ORFs of the group were regarded as redundant sequences. ORFs with a length less than 100 bp were subsequently filtered out. Finally, a gene catalog containing 793,847 non-redundant genes was constructed, covering 77.4 ± 0.3% (mean ± s.e.m.) of the high-quality reads in each sample.

Based on this reference gene set, we carried out taxonomical assignment and functional annotation using the NR database (v3) and the KEGG database (release 59.0). In this study, 80% of the genes in the catalog could be robustly assigned to the NR database, the remaining genes were likely to be from currently undefined microbial species. At the functional level, we identified 4846 KEGG orthologues (KOs), covering 46.43% of the genes in the catalog.

### Taxonomical assignment and functional classification

The nucleotide sequences of predicted genes were translated into protein sequences using the NCBI Genetic Codes 11. BLASTp was employed to conduct the taxonomical assignment and functional classification of predicted genes against the NR database (v3) and KEGG database (release 59.0) with *E* value ≤1 × 10^−3^. All genes were searched against IMG (v3.4) with BALSTN using default parameters except that the *E* value was set to 1 × 10^−5^. The taxonomical association of a gene was decided by the lowest common ancestor of all its taxonomical annotation results. Genes annotated by KEGG were assigned to KEGG pathways. In total, we identified 4846 KOs in the reference gene set with 80.0 and 46.4% genes of reference gene set having taxonomical and functional information, respectively.

### Relative abundance of genes and KOs

The high-quality clean paired-end reads from each sample were aligned against the reference genes set by *SOAP2* using a criterion requiring an identity >90%. We only counted the number of reads which fulfilled the following criteria: (i) Paired-end reads could be mapped onto the gene sequence with a moderate insert-size; (ii) One of the paired-end reads could be mapped onto the end of gene sequence, while the other reads mapped outside the gene region. In both situations, the mapped paired-end reads were counted as one copy. The number of reads mapped onto a certain gene was normalized by the gene length. Subsequently, the relative abundance table was constructed by normalizing the sum of all genes of a sample to 1. In Eq. (1), ${x}_i$ designates the number of reads that mapped onto a certain gene, and *L*_*i*_ the length of that gene, then the relative abundance of that gene ${a}_i$ equals to

1 $$ {a}_i=\frac{\frac{x_i}{L_i}}{{\displaystyle {\sum}_j}\frac{x_j}{L_j}} $$

where $j$ runs through the whole reference gene set.

All samples were treated in the same way resulting in a table containing the relative abundance of all genes of all samples. The KO profile was derived by summing up the relative abundance of genes which aligned to the same KO. As the same gene could belong to more than one KO group, we included the relative abundance of these genes in all the KO’s.

### Biodiversity analysis

Shannon index and Sørensen-Dice similarity index were calculated based on the relative abundance table of the 54 samples.

### Statistical analysis

All statistical analyses were implemented by the R software. Principal coordinates analysis (PCoA) was implemented using the “ade4” package [46]. To equalize the relative importance of common and rare genes/species/KOs, square root transformation was performed before PCA. Wilcoxon rank-sum test was employed to do the comparison analysis. Pair-wise Wilcoxon rank-sum test was used to do the difference comparison among experimental groups, in which the Hommel’s method was used to counteract the problem of multiple comparisons [47].

2 $$ \frac{\left({\mathrm{Abund}}_i-{\mathrm{Abund}}_j\right)}{{\mathrm{Abund}}_j} \times 100\% $$

The definition of effect size used in functional analysis is demonstrated in the Eq. (2), where ${\mathrm{Abund}}_i$ is the average function abundance of experimental group $i$. Pearson correlation coefficient was used to measure the correlation between two features. The significance level was set to 0.05.

## Additional files

Additional file 1: Table S1. The metagenome reference set. (XLS 42 kb)

Additional file 2: Figure S1. Rarefaction curve based on gene profiles of the total set of 54 samples. The rarefaction curves showed saturation at the current sample scale. Completeness of the total sample set according to ICE (Incidence-based Coverage Estimator) and Chao1 indices was 99.5%. (PDF 923 kb)

Additional file 3: Figure S2. PCoA analysis including all samples based on (a) KEGG profile and (b) genus profile. Strain and diet strongly drive the separation at the KO level, but have less impact at the genus level. The empty, half-filled and full-filled points correspond to mice characterized as “lean”, or with “no significant increase in adipose tissue mass (NSI)”, and “significant increase adipose tissue mass (SI)”. (PDF 203 kb)

Additional file 4: Figure S3. Relative abundance of KOs in relation to mouse strain and diet. Most of the annotated KOs is involved in Metabolism, with diet having a strong influence on the relative abundance of KOs. Mice fed the LF diet had higher abundance of KOs involved in Metabolism, Genetic Information Processing and Organismal Systems, while gut microbiomes of mice fed the HF diet exhibited a higher abundance of KOs involved in Environmental Information Processing and Cellular processes. Statistical differences were analyzed by unpaired Wilcoxon Rank-Sum test (with FDR correction). Statistically significant differences (P < 0.05) between groups are denoted with different letters (a, b, c, d) on the top of the graphic boxes. (PDF 883 kb)

Additional file 5: Figure S4. Relative abundance of top 5 genera in relation to mouse strain and diet. In mice fed the HF diet, the abundance of *Bacteroides* decreased, whereas the abundance of *Clostridium*, *Roseburia*, and *Lactobacillus* increased. Irrespective of feed, *Bacteroides*, *Clostridium* and *Roseburia* were more abundant Sv129 mice than in BL6 mice, whereas the relative abundance of *Akkermansia* was higher in BL6 mice than in Sv129 mice. The statistical differences were analyzed by unpaired Wilcoxon Rank-Sum test (with FDR correction). Statistically significant differences (*P* < 0.05) between groups are denoted with different letters (a, b, c, d) on the top of the graphic boxes. (PDF 898 kb)

Additional file 6: Table S2. The *P* value of the PERMANOVA test. (XLS 10 kb)

Additional file 7: Figure S5. Alpha diversity based on (a) genus and (b) KO profiles. At the genus level, HF feeding increased alpha diversity in both Sv129 and BL6, while at the KO level HF feeding did not increase alpha diversity relative to LF feeding. Comparison between mouse strains showed that alpha diversity at the KO level in HF-fed mice was higher in BL6 mice than in Sv129 mice. Statistic differences were analyzed by unpaired Wilcoxon Rank-Sum test (with FDR correction). Statistically significant differences (*P* < 0.05) between groups are denoted with different letters (a, b, c, d) on the top of the graphic boxes. (PDF 892 kb)

Additional file 8: FigureS6. Gene distribution in Sv129 and BL6 mice fed LF and HF diets. In mice fed the LF diet, 60.18% of the genes were identical in both strains, 27.47% of the genes were enriched in Sv129 mice and 12.36% were enriched in the BL6 mice. When the diet was changed from LF to HF, most (72.09%) of the LF shared genes were still shared, and 60.46% of the genes which were enriched in LF-fed Sv129 mice were now shared by the Sv129 and BL6 mice. About half (49.4%) of the genes enriched in BL6 mice fed the LF diet were still selectively enrichedafter the mice had been fed the HF diet. (PDF 195 kb)

Additional file 9: Figure S7. Relative abundance of the top 5 phyla in relation to mouse strain and diet. In keeping with previous studies, HF feeding caused a marked decrease in the relative abundance of *Bacteroidetes* and an increase in the relative abundance of *Firmicutes* in both strains. The relative abundance of *Verrucomicrobia* was significantly higher in BL6 mice than in Sv129 mice, irrespective of the diet. Statistical differences were analyzed by unpaired Wilcoxon Rank-Sum test (with FDR correction). Statistically significant differences (P < 0.05) between groups are denoted with different letters (a, b, c, d) on the top of the graphic boxes. (PDF 193 kb)

Additional file 10: Figure S8. Relative abundance of low abundant phyla in relation to mouse strain and diet. Thefigure shows phyla whose relative abundances were lower than 1%. Marked effects of diets were also observed in these phyla. Statistical differences were analyzed by unpaired Wilcoxon Rank-Sum test (with FDR correction). Statistically significant differences (*P* < 0.05) between groups are denoted with different letters (a, b, c, d) on the top of the graphic boxes. (PDF 927 kb)

Additional file 11: Figure S9. Relative abundance of very low abundant genera in relation to mouse strain and diet. The figure shows genera whose relative abundances were lower than 0.1%. Marked effects of diets were also observed in these low abundant genera. Statistical differences were analyzed by unpaired Wilcoxon Rank-Sum test (with FDR correction). Statistically significant differences (*P* < 0.05) between groups are denoted with different letters (a, b, c, d) on the top of the graphic boxes. (PDF 951 kb)

Additional file 12: Figure S10. The top 5 most abundant annotated species in in relation to mouse strain and diet. The figure shows the top 5 most abundant annotated species, which also clearly exhibited significant changes in abundance in relation to diet. Statistical differences were analyzed by unpaired Wilcoxon Rank-Sum test (with FDR correction). Statistically significant differences (*P* < 0.05) between groups are denoted with different letters (a, b, c, d) on the top of the graphic boxes. (PDF 913 kb)

Additional file 13: Figure S11. Low abundant annotated species in relation to mouse strain and diet. The figure shows low abundant annotated species, which also clearly showed significant changes in abundance in relation to diet. Statistical differences were analyzed by unpaired Wilcoxon Rank-Sum test (with FDR correction). Statistically significant differences (*P* < 0.05) between groups are denoted with different letters (a, b, c, d) on the top of the graphic boxes. (PDF 948 kb)

Additional file 14: Figure S12. Distribution of the KOs involved in butyrate formation module in relation to mouse strain and diet. The relative abundance of enzymes involved in butyrate formation was calculated at the KO level. The majority of the KOs exhibited a higher abundance in Sv129 mice compared with BL6 mice, irrespective of the diet. Statistical differences were analyzed by unpaired Wilcoxon Rank-Sum test (with FDR correction). Statistically significant differences (*P* < s0.05) between groups are denoted with different letters (a, b, c, d) on the top of the graphic boxes. (PDF 941 kb)

Additional file 15: Table S3. Strain specific fucntion changes responding to high-fat diet. (XLS 26 kb)

Additional file 16: Table S4. Diet-induced changes in pathway. (XLS 9 kb)

Additional file 17: Table S5. Diet-induced changes in module. (XLS 8 kb)

Additional file 18: Table S6. Consistence of HF enrichment and existence of glycerol kinase. (XLS 43 kb)

Additional file 19: Figure S13. Relative abundance of bile acid metabolizing bacteria. *Clostridium hylemonae*, *Clostridium leptum*, *Clostridium scindens*, and *Clostridium hiranonis* have been associated with bile acid metabolism. The relative abundance of these 4 species in relation to mouse strain and diet was calculated. All were found to be enriched in mice fed the HF. No effect of indomethacin supplementation was observed. Statistical differences were analyzed by unpaired Wilcoxon Rank-Sum test (with FDR correction). Statistically significant differences (*P* < 0.05) between groups are denoted with different letters (a, b, c, d) on the top of the graphic boxes. (PDF 907 kb)

Additional file 20: Figure S14. Relative abundance of Enterococcus faecalis-related bacteria and segmented filamentous bacteria. The abundance of Enterococcus faecalis-related bacteria and segmented filamentous bacteria has been reported to be affected by administration of indomethacin. The relative abundance of these bacteria was increased in HF-fed mice of both strain, but no effect of indomethacin was observed. Statistical differences were analyzed by unpaired Wilcoxon Rank-Sum test (with FDR correction). Statistically significant differences (*P* < 0.05) between groups are denoted with different letters (a, b, c, d) on the top of the graphic boxes. (PDF 969 kb)

## Abbreviations

BL6: C57BL/6JBomTac
COX: Cyclooxygenase
HF: High fat
HFI: HF diet supplemented with indomethacin
LF: Low fat
SCFAs: Short chain fatty acids
Sv129: 129S6/SvEvTac
WAT: White adipose tissue
WGS: Whole genome sequencing
