# Supplementary material for: High-fat feeding rather than obesity drives taxonomical and functional changes in the gut microbiota in mice
Source: Microbiome. 2017 Apr 8;5:43. doi: 10.1186/s40168-017-0258-6 (PMC5385073; doi:10.1186/s40168-017-0258-6)
Supplement: Supplementary file 2 — Rarefaction curve based on gene profiles of the total set of 54 samples. The rarefaction curves showed saturation at the current sample scale. Completeness of the total sample set according to ICE (Incidence-based Coverage Estimator) and Chao1 indices was 99.5%. (PDF 923 kb) [file 40168_2017_258_MOESM2_ESM.pdf]

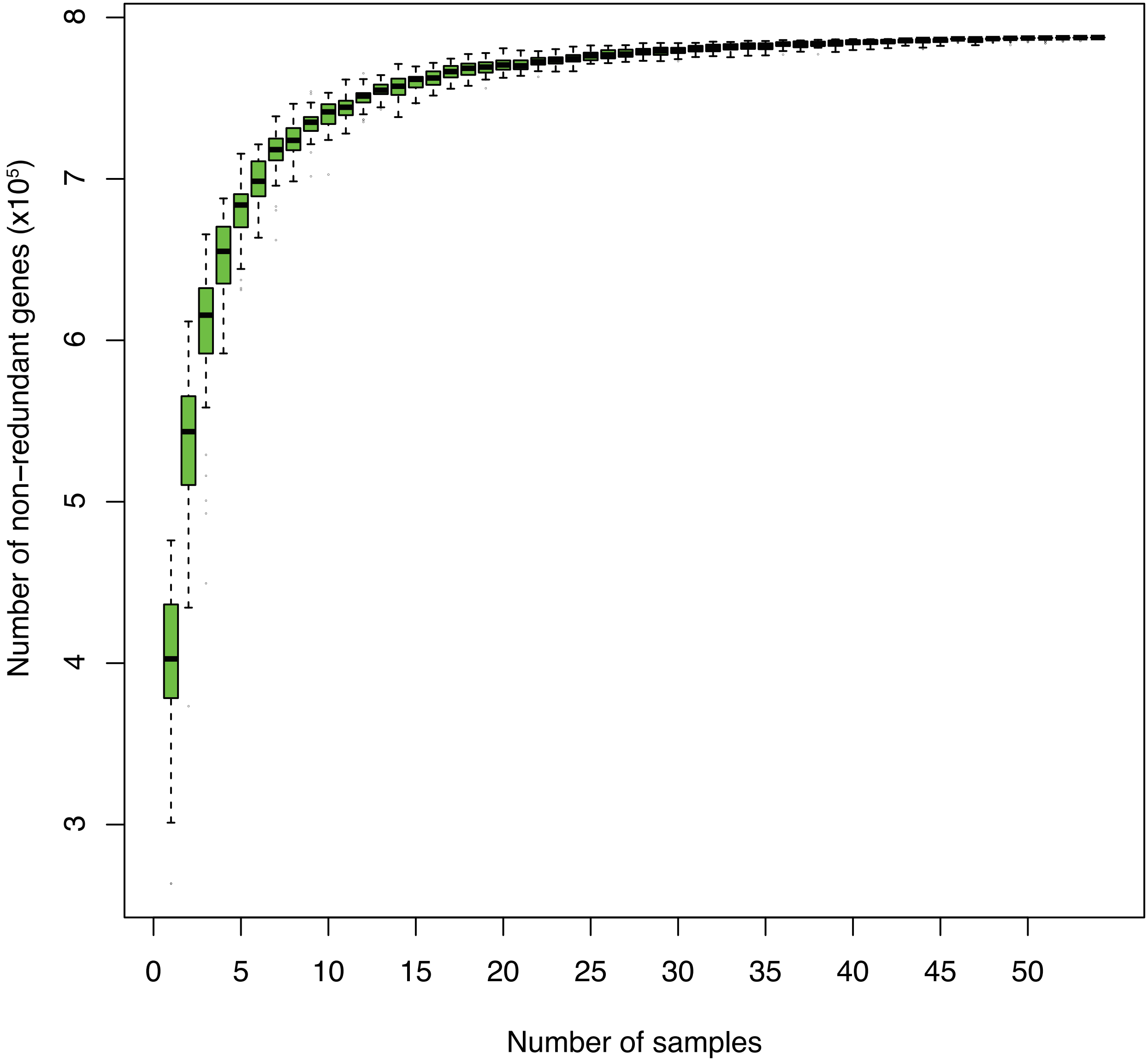

Figure S1. Rarefaction curve based on gene profiles of the total set of 54 samples.
The rarefaction curves showed saturation at the current sample scale. Completeness of the total sample set according to ICE (Incidence-based Coverage Estimator) and Chao1 indices was 99.5%.
