# Supplementary material for: High-fat feeding rather than obesity drives taxonomical and functional changes in the gut microbiota in mice
Source: Microbiome. 2017 Apr 8;5:43. doi: 10.1186/s40168-017-0258-6 (PMC5385073; doi:10.1186/s40168-017-0258-6)
Supplement: Supplementary file 3 — PCoA analysis including all samples based on (a) KEGG profile and (b) genus profile. Strain and diet strongly drive the separation at the KO level, but have less impact at the genus level. The empty, half-filled and full-filled points correspond to mice characterized as “lean”, or with “no significant increase in adipose tissue mass (NSI)”, and “significant increase adipose tissue mass (SI)”. (PDF 203 kb) [file 40168_2017_258_MOESM3_ESM.pdf]

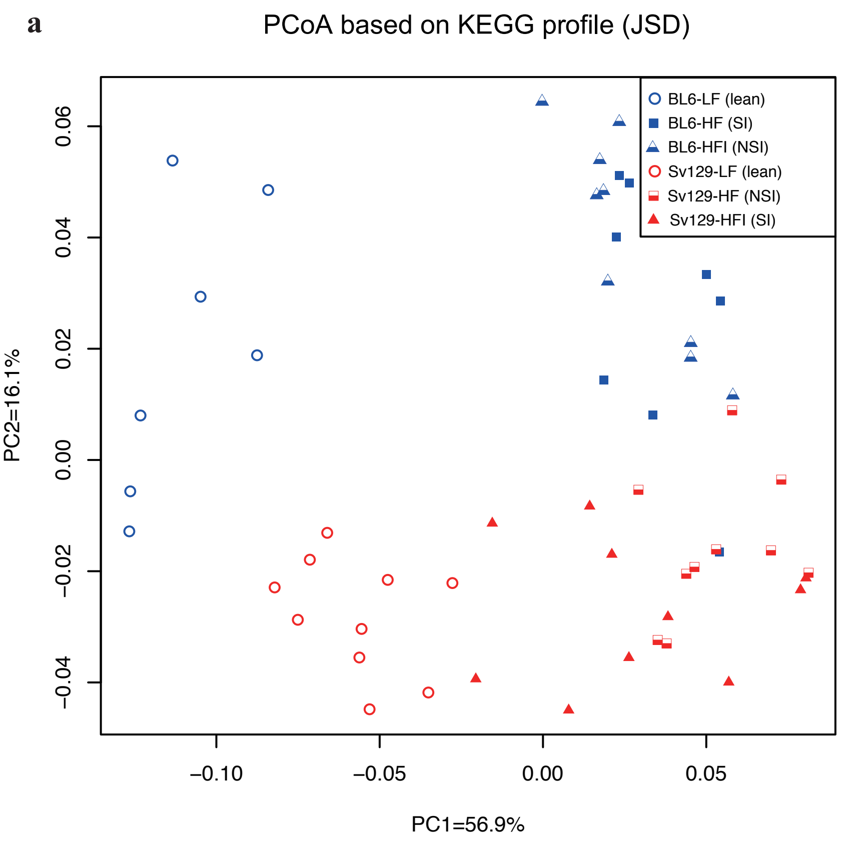

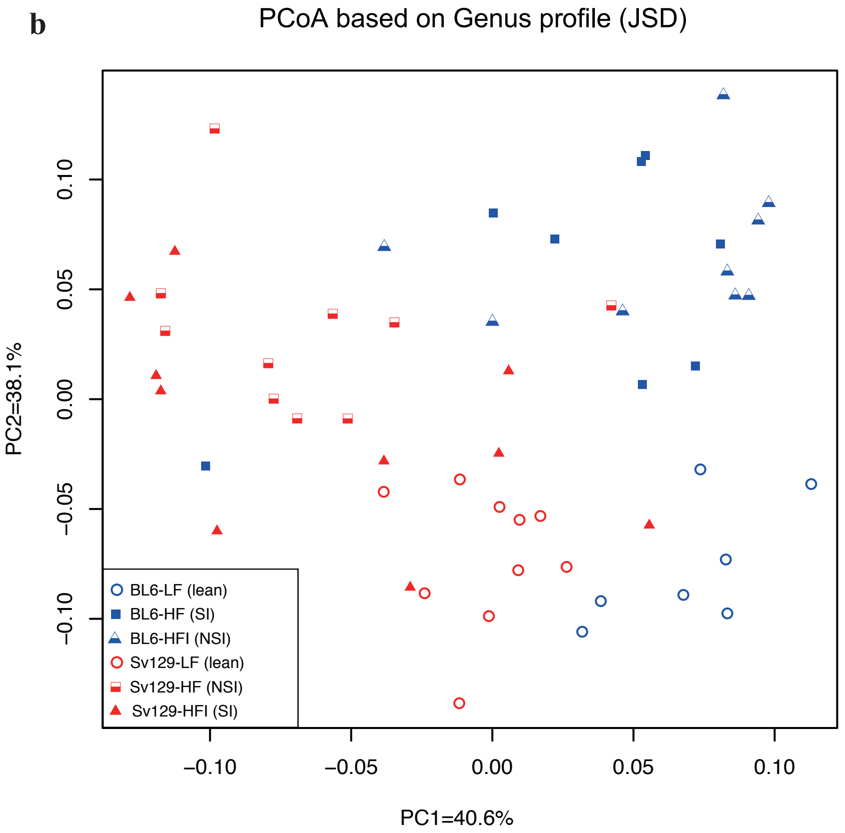

Figure S2. PCoA analysis including all samples based on (a) KEGG profile and (b) genus profile. Strain and diet strongly drive the separation at the KO level, but have less impact at the genus level. The empty, half-filled and full-filled points correspond to mice characterized as "lean", or with "no significant increase in adipose tissue mass (NSI)", and "significant increase adipose tissue mass (SI)".
