# Supplementary material for: High-fat feeding rather than obesity drives taxonomical and functional changes in the gut microbiota in mice
Source: Microbiome. 2017 Apr 8;5:43. doi: 10.1186/s40168-017-0258-6 (PMC5385073; doi:10.1186/s40168-017-0258-6)
Supplement: Supplementary file 4 — Relative abundance of KOs in relation to mouse strain and diet. Most of the annotated KOs is involved in Metabolism, with diet having a strong influence on the relative abundance of KOs. Mice fed the LF diet had higher abundance of KOs involved in Metabolism, Genetic Information Processing and Organismal Systems, while gut microbiomes of mice fed the HF diet exhibited a higher abundance of KOs involved in Environmental Information Processing and Cellular processes. Statistical differences were analyzed by unpaired Wilcoxon Rank-Sum test (with FDR correction). Statistically significant differences (P < 0.05) between groups are denoted with different letters (a, b, c, d) on the top of the graphic boxes. (PDF 883 kb) [file 40168_2017_258_MOESM4_ESM.pdf]

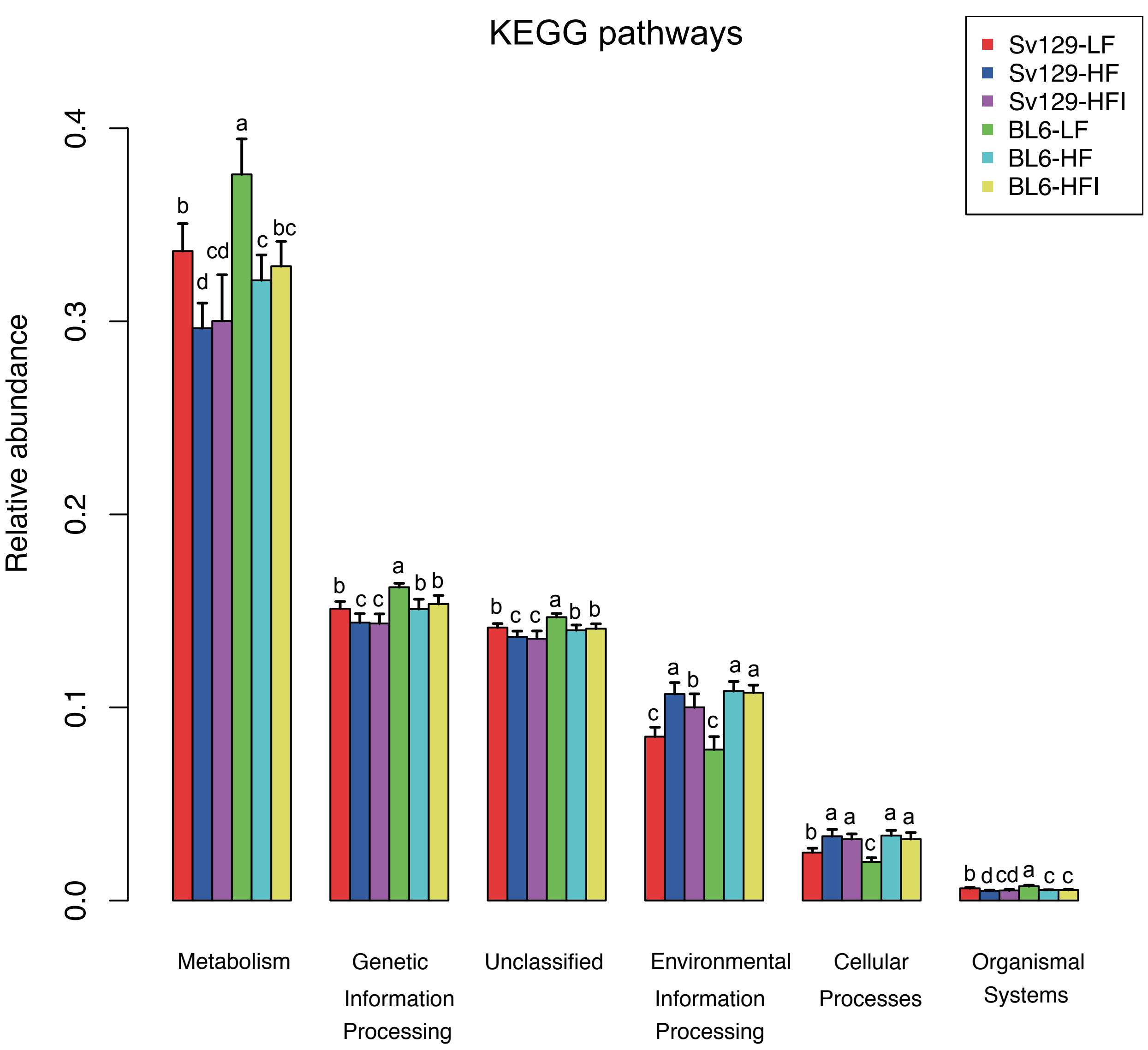

Figure S3. Relative abundance of KOs in relation to mouse strain and diet. Most of the annotated KOs is involved in Metabolism, with diet having a strong influence on the relative abundance of KOs. Mice fed the LF diet had higher abundance of KOs involved in Metabolism, Genetic Information Processing and Organismal Systems, while gut microbiomes of mice fed the HF diet exhibited a higher abundance of KOs involved in Environmental Information Processing and Cellular processes. Statistical differences were analyzed by unpaired Wilcoxon Rank-Sum test (with FDR correction). Statistically significant differences ($P < 0.05$) between groups are denoted with different letters (a, b, c, d) on the top of the graphic boxes.
