# Supplementary material for: High-fat feeding rather than obesity drives taxonomical and functional changes in the gut microbiota in mice
Source: Microbiome. 2017 Apr 8;5:43. doi: 10.1186/s40168-017-0258-6 (PMC5385073; doi:10.1186/s40168-017-0258-6)
Supplement: Supplementary file 5 — Relative abundance of top 5 genera in relation to mouse strain and diet. In mice fed the HF diet, the abundance of Bacteroides decreased, whereas the abundance of Clostridium, Roseburia, and Lactobacillus increased. Irrespective of feed, Bacteroides, Clostridium and Roseburia were more abundant Sv129 mice than in BL6 mice, whereas the relative abundance of Akkermansia was higher in BL6 mice than in Sv129 mice. The statistical differences were analyzed by unpaired Wilcoxon Rank-Sum test (with FDR correction). Statistically significant differences (P < 0.05) between groups are denoted with different letters (a, b, c, d) on the top of the graphic boxes. (PDF 898 kb) [file 40168_2017_258_MOESM5_ESM.pdf]

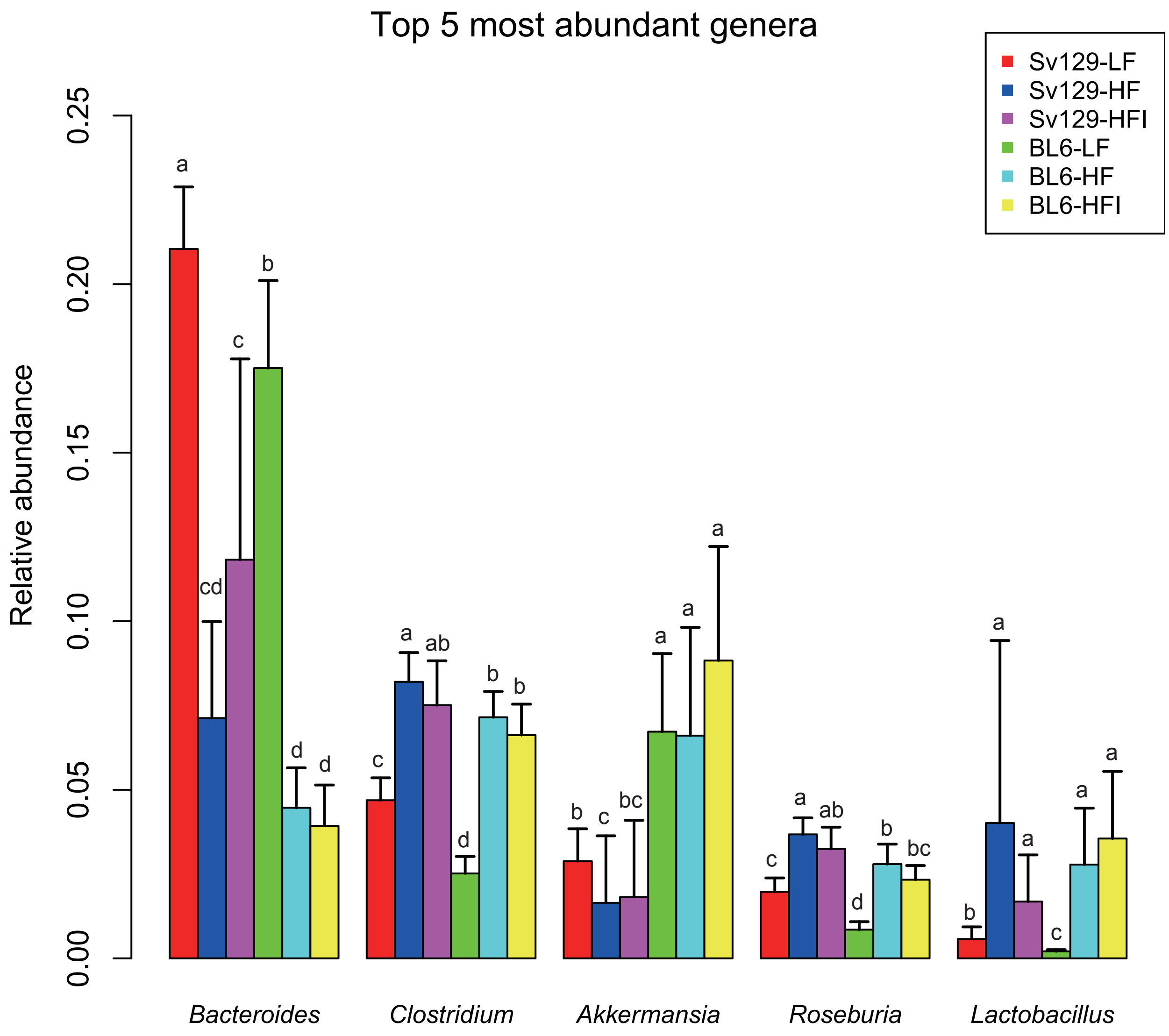

Figure S4. Relative abundance of top 5 genera in relation to mouse strain and diet. In mice fed the HF diet, the abundance of *Bacteroides* decreased, whereas the abundance of *Clostridium*, *Roseburia* and *Lactobacillus* increased. Irrespective of feed, *Bacteroides*, *Clostridium* and *Roseburia* were more abundant Sv129 mice than in BL6 mice, whereas the relative abundance of *Akkermansia* was higher in BL6 mice than in Sv129 mice. The statistical differences were analyzed by unpaired Wilcoxon Rank-Sum test (with FDR correction). Statistically significant differences ($P < 0.05$) between groups are denoted with different letters (a, b, c, d) on the top of the graphic boxes.
