# Supplementary material for: High-fat feeding rather than obesity drives taxonomical and functional changes in the gut microbiota in mice
Source: Microbiome. 2017 Apr 8;5:43. doi: 10.1186/s40168-017-0258-6 (PMC5385073; doi:10.1186/s40168-017-0258-6)
Supplement: Supplementary file 7 — Alpha diversity based on (a) genus and (b) KO profiles. At the genus level, HF feeding increased alpha diversity in both Sv129 and BL6, while at the KO level HF feeding did not increase alpha diversity relative to LF feeding. Comparison between mouse strains showed that alpha diversity at the KO level in HF-fed mice was higher in BL6 mice than in Sv129 mice. Statistic differences were analyzed by unpaired Wilcoxon Rank-Sum test (with FDR correction). Statistically significant differences (P < 0.05) between groups are denoted with different letters (a, b, c, d) on the top of the graphic boxes. (PDF 892 kb) [file 40168_2017_258_MOESM7_ESM.pdf]

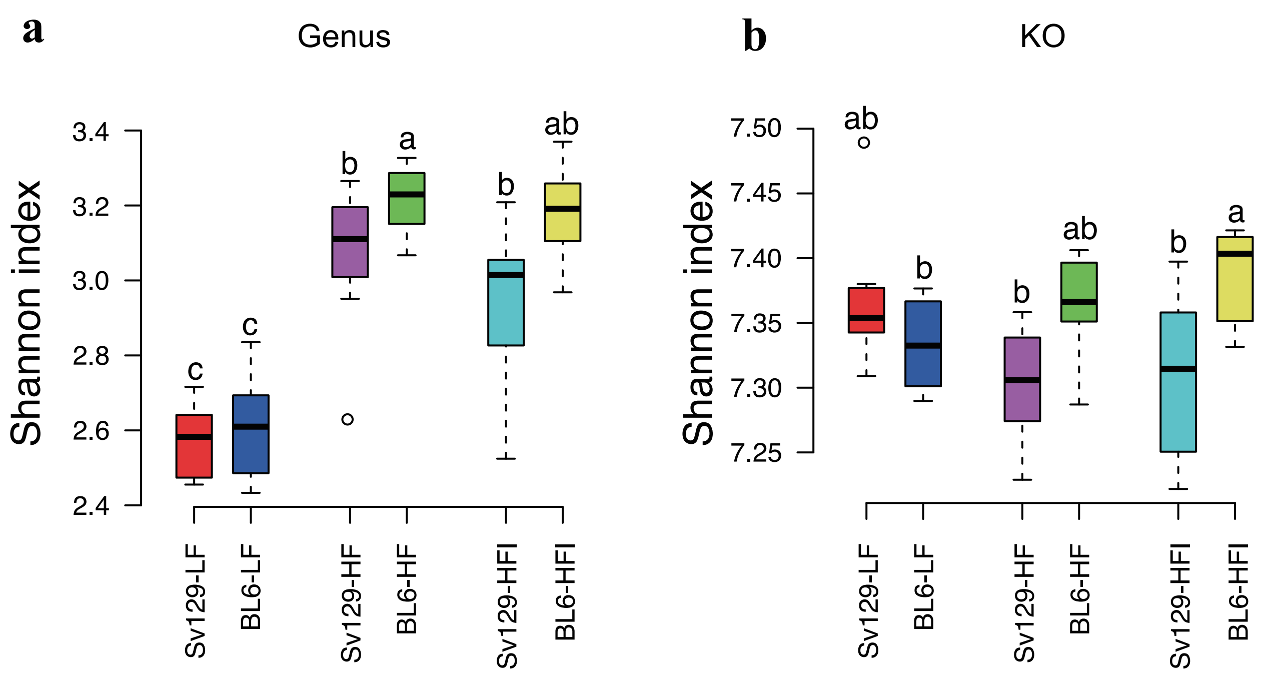

Figure S5. Alpha diversity based on (a) genus and (b) KO profiles. At the genus level, HF feeding increased alpha diversity in both Sv129 and BL6, while at the KO level HF feeding did not increase alpha diversity relative to LF feeding. Comparison between mouse strains showed that alpha diversity at the KO level in HF fed mice was higher in BL6 mice than in Sv129 mice. Statistic differences were analyzed by unpaired Wilcoxon Rank-Sum test (with FDR correction). Statistically significant differences ($P < 0.05$) between groups are denoted with different letters (a, b, c, d) on the top of the graphic boxes.
