# Supplementary material for: High-fat feeding rather than obesity drives taxonomical and functional changes in the gut microbiota in mice
Source: Microbiome. 2017 Apr 8;5:43. doi: 10.1186/s40168-017-0258-6 (PMC5385073; doi:10.1186/s40168-017-0258-6)
Supplement: Supplementary file 8 — Gene distribution in Sv129 and BL6 mice fed LF and HF diets. In mice fed the LF diet, 60.18% of the genes were identical in both strains, 27.47% of the genes were enriched in Sv129 mice and 12.36% were enriched in the BL6 mice. When the diet was changed from LF to HF, most (72.09%) of the LF shared genes were still shared, and 60.46% of the genes which were enriched in LF-fed Sv129 mice were now shared by the Sv129 and BL6 mice. About half (49.4%) of the genes enriched in BL6 mice fed the LF diet were still selectively enrichedafter the mice had been fed the HF diet. (PDF 195 kb) [file 40168_2017_258_MOESM8_ESM.pdf]

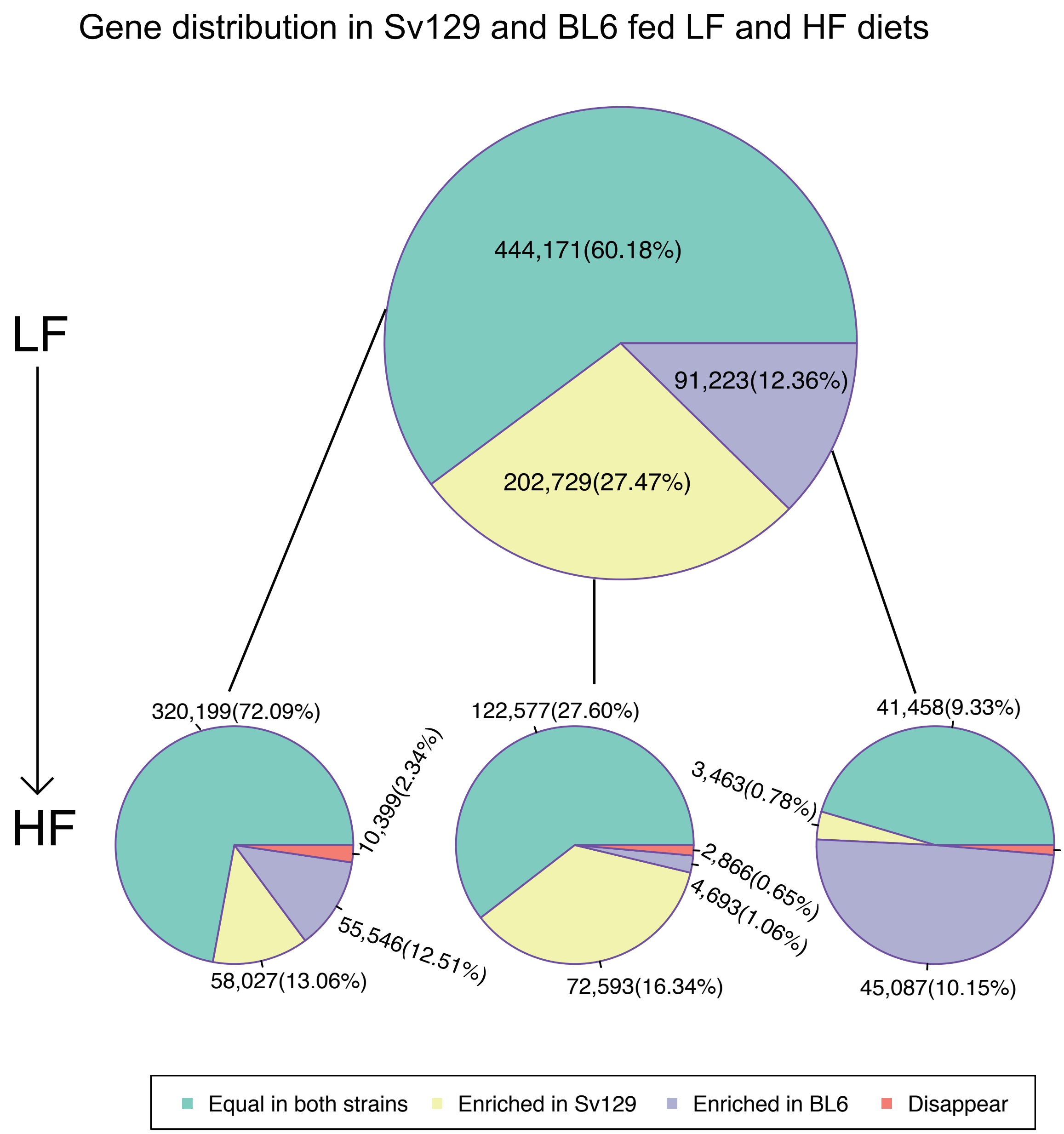

Figure S6. Gene distribution in Sv129 and BL6 mice fed LF and HF diets. In mice fed the LF diet, 60.18% of the genes were identical in both strains, 27.47% of the genes were enriched in Sv129 mice and 12.36% were enriched in the BL6 mice. When the diet was changed from LF to HF, most (72.09%) of the LF shared genes were still shared, and 60.46% of the genes which were enriched in LF fed Sv129 mice were now shared by the Sv129 and BL6 mice. About half (49.4%) of the genes enriched in BL6 mice fed the LF diet were still selectively enrichedafter the mice had been fed the HF diet.
