# Supplementary material for: High-fat feeding rather than obesity drives taxonomical and functional changes in the gut microbiota in mice
Source: Microbiome. 2017 Apr 8;5:43. doi: 10.1186/s40168-017-0258-6 (PMC5385073; doi:10.1186/s40168-017-0258-6)
Supplement: Supplementary file 9 — Relative abundance of the top 5 phyla in relation to mouse strain and diet. In keeping with previous studies, HF feeding caused a marked decrease in the relative abundance of Bacteroidetes and an increase in the relative abundance of Firmicutes in both strains. The relative abundance of Verrucomicrobia was significantly higher in BL6 mice than in Sv129 mice, irrespective of the diet. Statistical differences were analyzed by unpaired Wilcoxon Rank-Sum test (with FDR correction). Statistically significant differences (P < 0.05) between groups are denoted with different letters (a, b, c, d) on the top of the graphic boxes. (PDF 193 kb) [file 40168_2017_258_MOESM9_ESM.pdf]

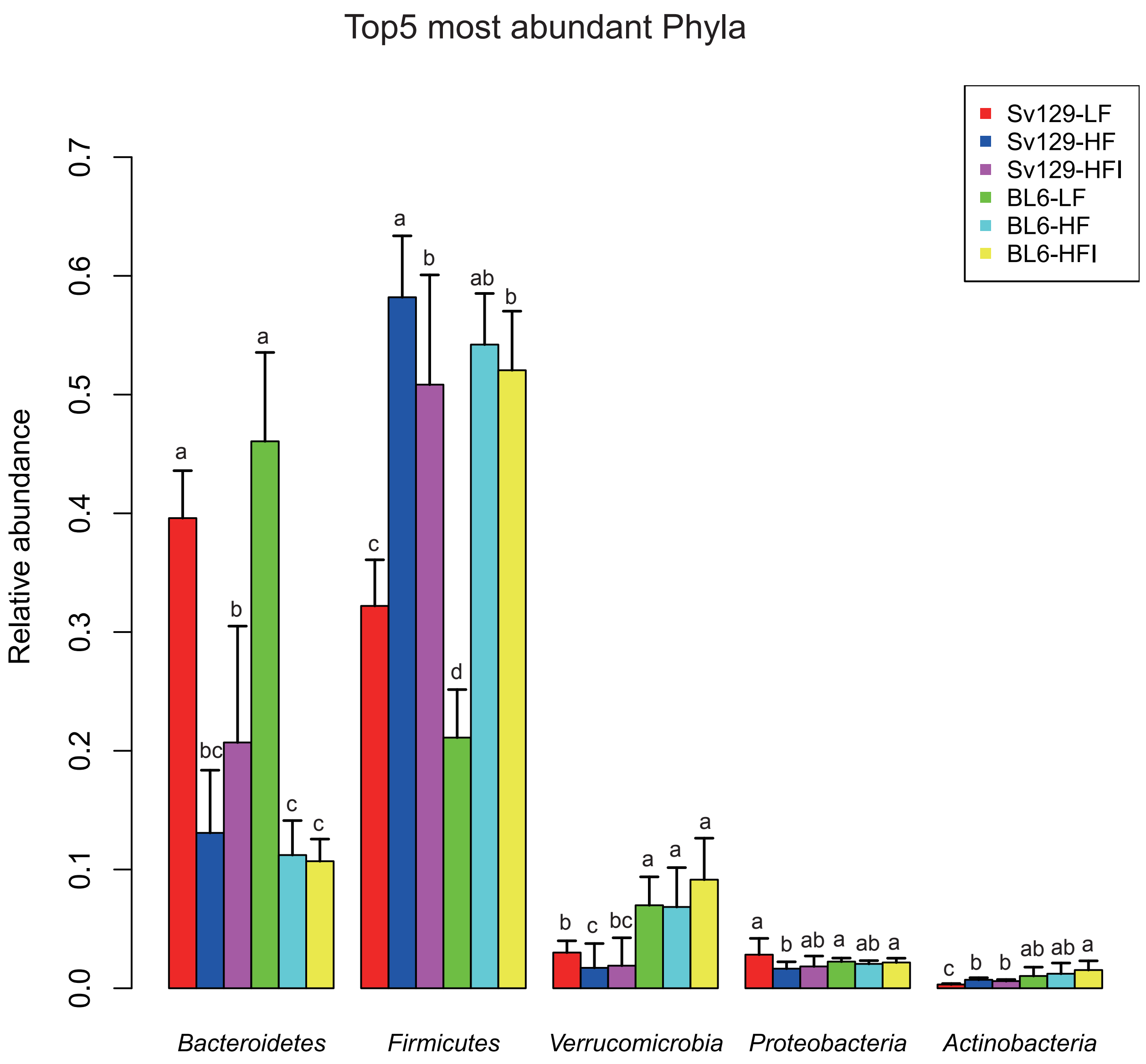

Figure S7. Relative abundance of the top 5 phyla in relation to mouse strain and diet. In keeping with previous studies, HF feeding caused a marked decrease in the relative abundance of *Bacteroidetes* and an increase in the relative abundance of *Firmicutes* in both strains. The relative abundance of *Verrucomicrobia* was significantly higher in BL6 mice than in Sv129 mice, irrespective of the diet. Statistical differences were analyzed by unpaired Wilcoxon Rank-Sum test (with FDR correction). Statistically significant differences ($P < 0.05$) between groups are denoted with different letters (a, b, c, d) on the top of the graphic boxes.
