# Supplementary material for: High-fat feeding rather than obesity drives taxonomical and functional changes in the gut microbiota in mice
Source: Microbiome. 2017 Apr 8;5:43. doi: 10.1186/s40168-017-0258-6 (PMC5385073; doi:10.1186/s40168-017-0258-6)
Supplement: Supplementary file 12 — The top 5 most abundant annotated species in in relation to mouse strain and diet. The figure shows the top 5 most abundant annotated species, which also clearly exhibited significant changes in abundance in relation to diet. Statistical differences were analyzed by unpaired Wilcoxon Rank-Sum test (with FDR correction). Statistically significant differences (P < 0.05) between groups are denoted with different letters (a, b, c, d) on the top of the graphic boxes. (PDF 913 kb) [file 40168_2017_258_MOESM12_ESM.pdf]

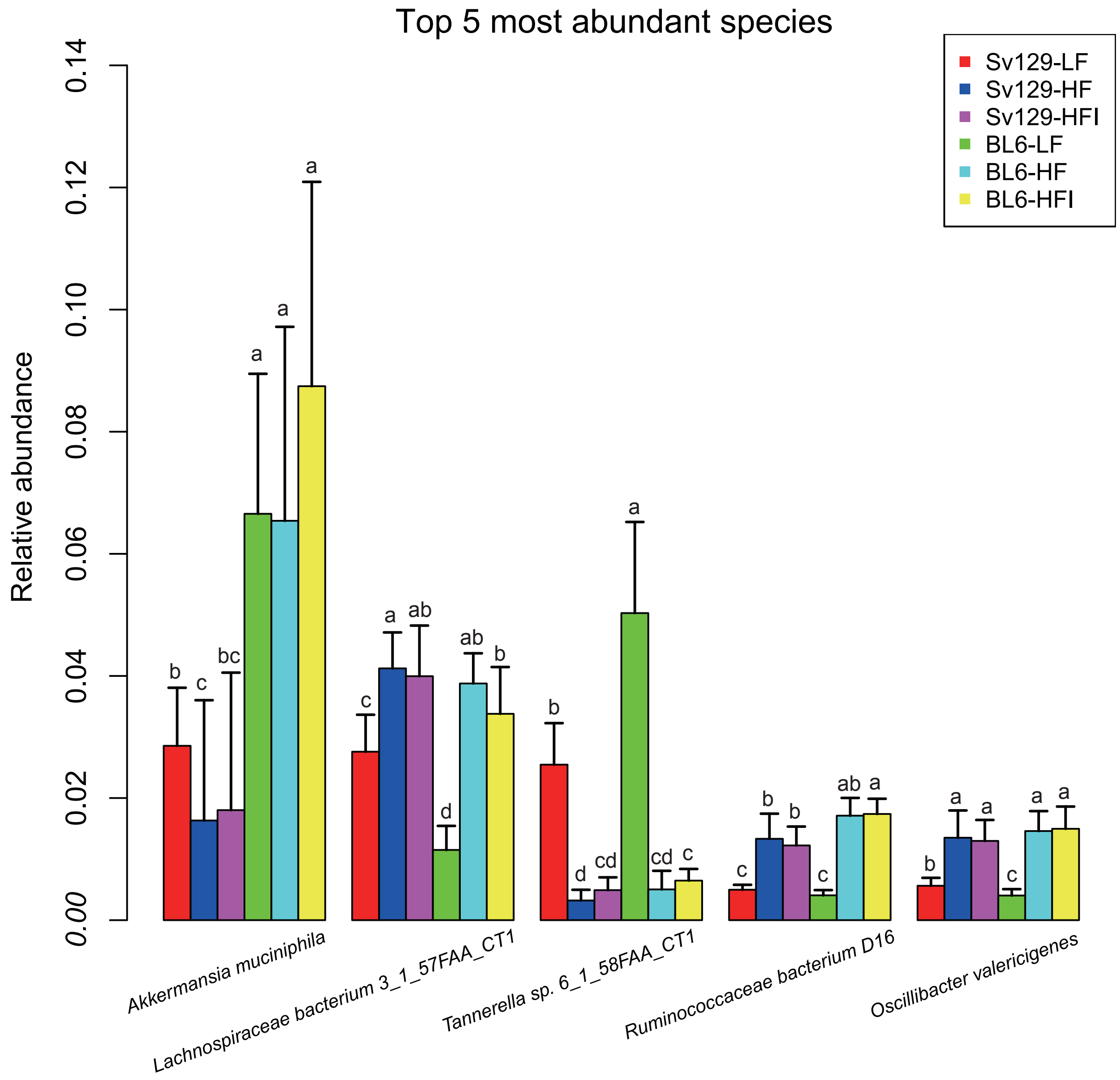

Figure S10. The top 5 most abundant annotated species in in relation to mouse strain and diet. The figure shows the top 5 most abundant annotated species, which also clearly exhibited significant changes in abundance in relation to diet. Statistical differences were analyzed by unpaired Wilcoxon Rank-Sum test (with FDR correction). Statistically significant differences ($P < 0.05$) between groups are denoted with different letters (a, b, c, d) on the top of the graphic boxes.
