# Supplementary material for: High-fat feeding rather than obesity drives taxonomical and functional changes in the gut microbiota in mice
Source: Microbiome. 2017 Apr 8;5:43. doi: 10.1186/s40168-017-0258-6 (PMC5385073; doi:10.1186/s40168-017-0258-6)
Supplement: Supplementary file 13 — Low abundant annotated species in relation to mouse strain and diet. The figure shows low abundant annotated species, which also clearly showed significant changes in abundance in relation to diet. Statistical differences were analyzed by unpaired Wilcoxon Rank-Sum test (with FDR correction). Statistically significant differences (P < 0.05) between groups are denoted with different letters (a, b, c, d) on the top of the graphic boxes. (PDF 948 kb) [file 40168_2017_258_MOESM13_ESM.pdf]

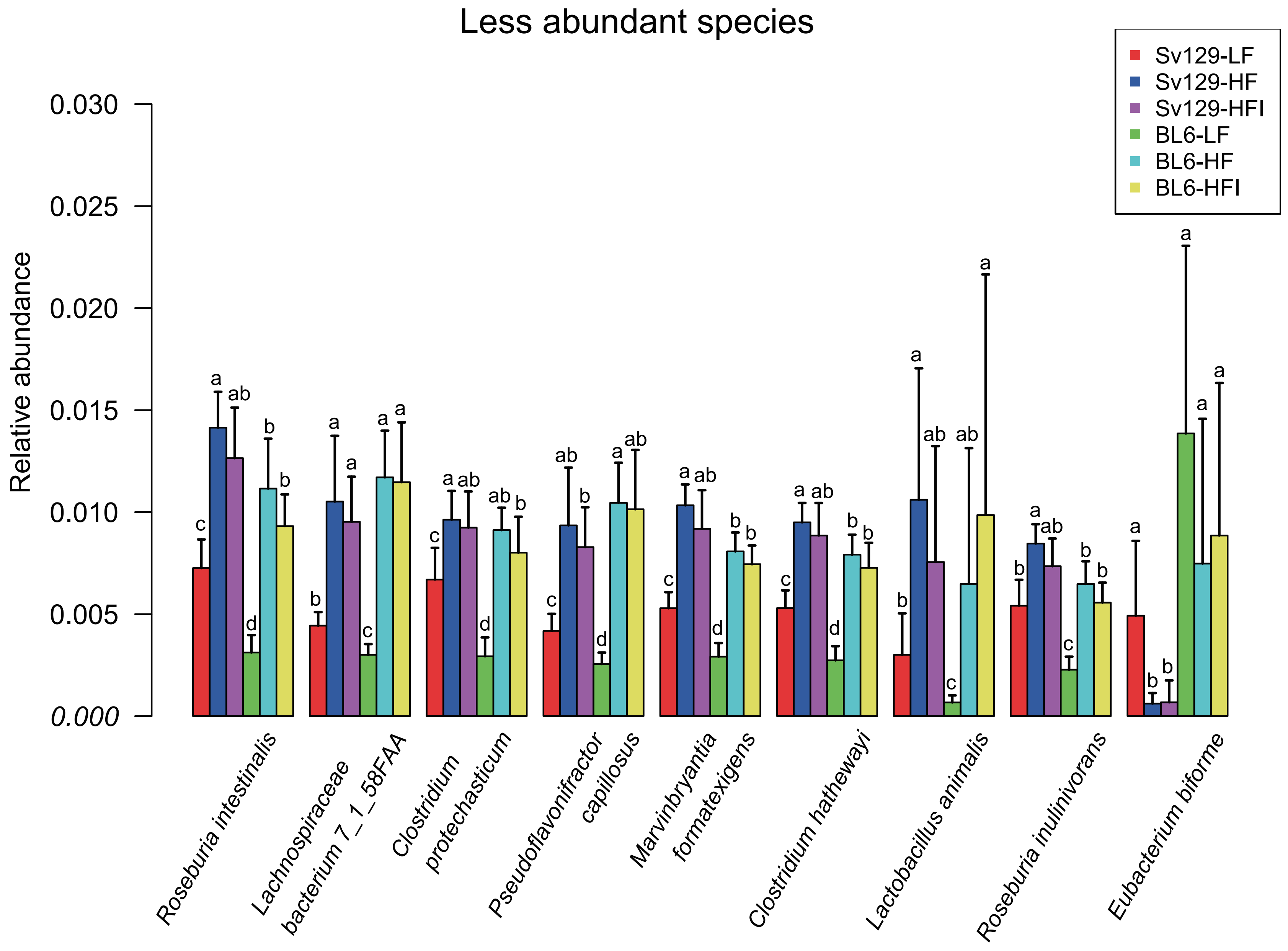

Figure S11. Low abundant annotated species in relation to mouse strain and diet. The figure shows low abundant annotated species, which also clearly showed significant changes in abundance in relation to diet. Statistical differences were analyzed by unpaired Wilcoxon Rank-Sum test (with FDR correction). Statistically significant differences ($P < 0.05$) between groups are denoted with different letters (a, b, c, d) on the top of the graphic boxes.
