# Supplementary material for: High-fat feeding rather than obesity drives taxonomical and functional changes in the gut microbiota in mice
Source: Microbiome. 2017 Apr 8;5:43. doi: 10.1186/s40168-017-0258-6 (PMC5385073; doi:10.1186/s40168-017-0258-6)
Supplement: Supplementary file 14 — Distribution of the KOs involved in butyrate formation module in relation to mouse strain and diet. The relative abundance of enzymes involved in butyrate formation was calculated at the KO level. The majority of the KOs exhibited a higher abundance in Sv129 mice compared with BL6 mice, irrespective of the diet. Statistical differences were analyzed by unpaired Wilcoxon Rank-Sum test (with FDR correction). Statistically significant differences (P < s0.05) between groups are denoted with different letters (a, b, c, d) on the top of the graphic boxes. (PDF 941 kb) [file 40168_2017_258_MOESM14_ESM.pdf]

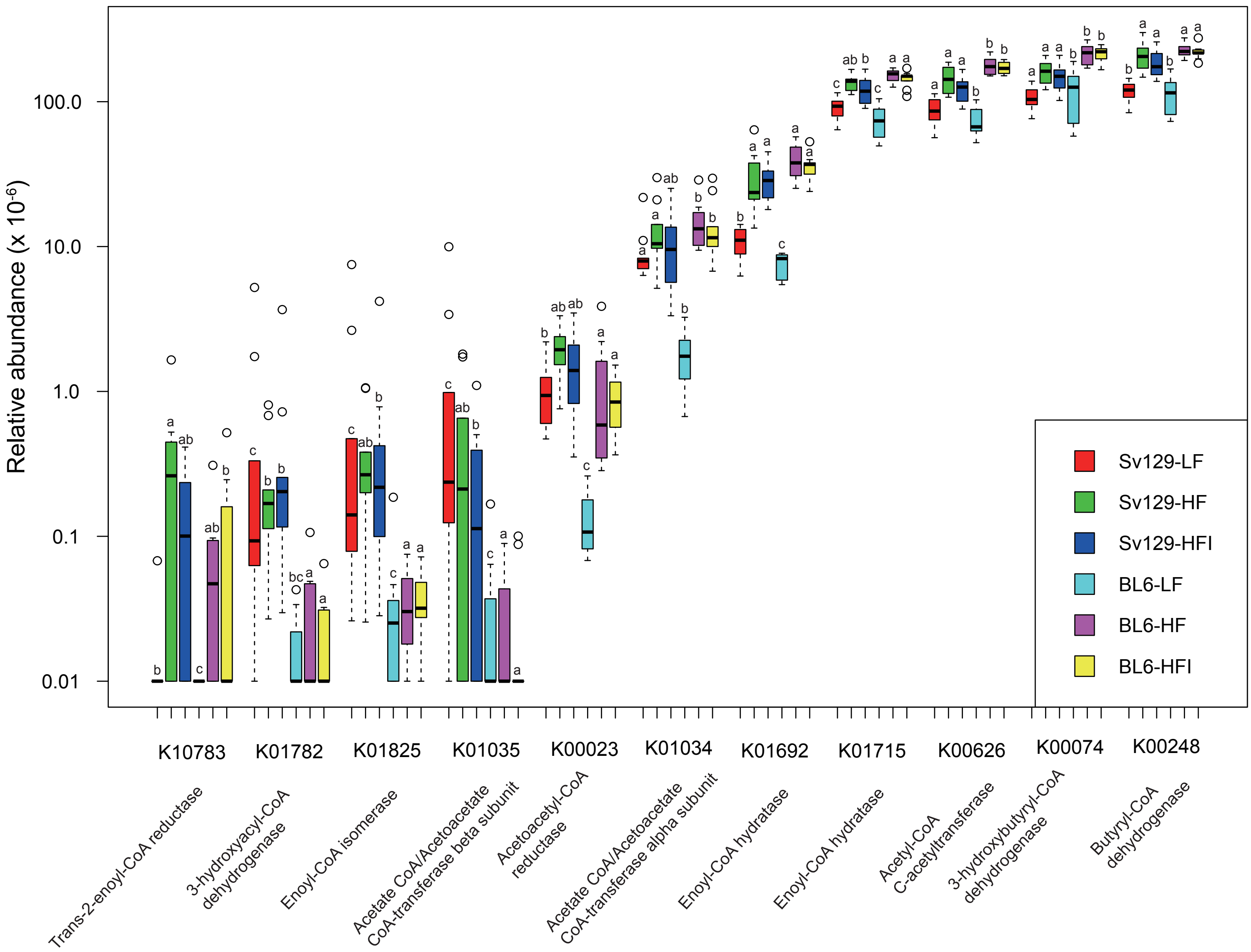

Figure S12. Distribution of the KOs involved in butyrate formation module in relation to mouse strain and diet. The relative abundance of enzymes involved in butyrate formation was calculated at the KO level. The majority of the KOs exhibited a higher abundance in Sv129 mice compared with BL6 mice, irrespective of the diet. Statistical differences were analyzed by unpaired Wilcoxon Rank-Sum test (with FDR correction). Statistically significant differences ($P < 0.05$) between groups are denoted with different letters (a, b, c, d) on the top of the graphic boxes.
