# Supplementary material for: High-fat feeding rather than obesity drives taxonomical and functional changes in the gut microbiota in mice
Source: Microbiome. 2017 Apr 8;5:43. doi: 10.1186/s40168-017-0258-6 (PMC5385073; doi:10.1186/s40168-017-0258-6)
Supplement: Supplementary file 19 — Relative abundance of bile acid metabolizing bacteria. Clostridium hylemonae, Clostridium leptum, Clostridium scindens, and Clostridium hiranonis have been associated with bile acid metabolism. The relative abundance of these 4 species in relation to mouse strain and diet was calculated. All were found to be enriched in mice fed the HF. No effect of indomethacin supplementation was observed. Statistical differences were analyzed by unpaired Wilcoxon Rank-Sum test (with FDR correction). Statistically significant differences (P < 0.05) between groups are denoted with different letters (a, b, c, d) on the top of the graphic boxes. (PDF 907 kb) [file 40168_2017_258_MOESM19_ESM.pdf]

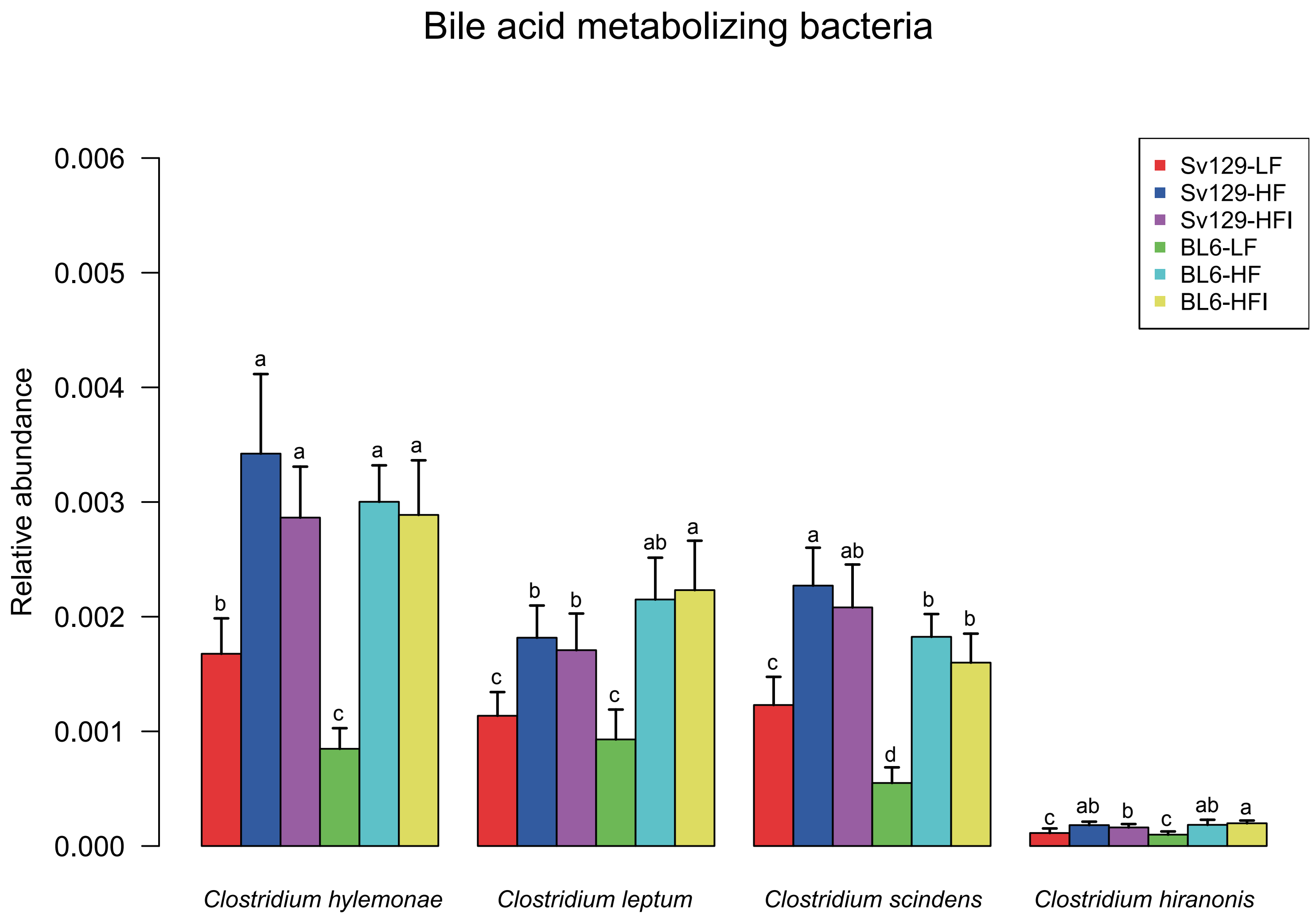

Figure S13. Relative abundance of bile acid metabolizing bacteria. *Clostridium hylemonae*, *Clostridium leptum*, *Clostridium scindens* and *Clostridium hiranonis* have been associated with bile acid metabolism. The relative abundance of these 4 species in relation to mouse strain and diet was calculated. All were found to be enriched in mice fed the HF. No effect of indomethacin supplementation was observed. Statistical differences were analyzed by unpaired Wilcoxon Rank-Sum test (with FDR correction). Statistically significant differences ($P < 0.05$) between groups are denoted with different letters (a, b, c, d) on the top of the graphic boxes.
