# Supplementary material for: High-fat feeding rather than obesity drives taxonomical and functional changes in the gut microbiota in mice
Source: Microbiome. 2017 Apr 8;5:43. doi: 10.1186/s40168-017-0258-6 (PMC5385073; doi:10.1186/s40168-017-0258-6)
Supplement: Supplementary file 20 — Relative abundance of Enterococcus faecalis-related bacteria and segmented filamentous bacteria. The abundance of Enterococcus faecalis-related bacteria and segmented filamentous bacteria has been reported to be affected by administration of indomethacin. The relative abundance of these bacteria was increased in HF-fed mice of both strain, but no effect of indomethacin was observed. Statistical differences were analyzed by unpaired Wilcoxon Rank-Sum test (with FDR correction). Statistically significant differences (P < 0.05) between groups are denoted with different letters (a, b, c, d) on the top of the graphic boxes. (PDF 969 kb) [file 40168_2017_258_MOESM20_ESM.pdf]

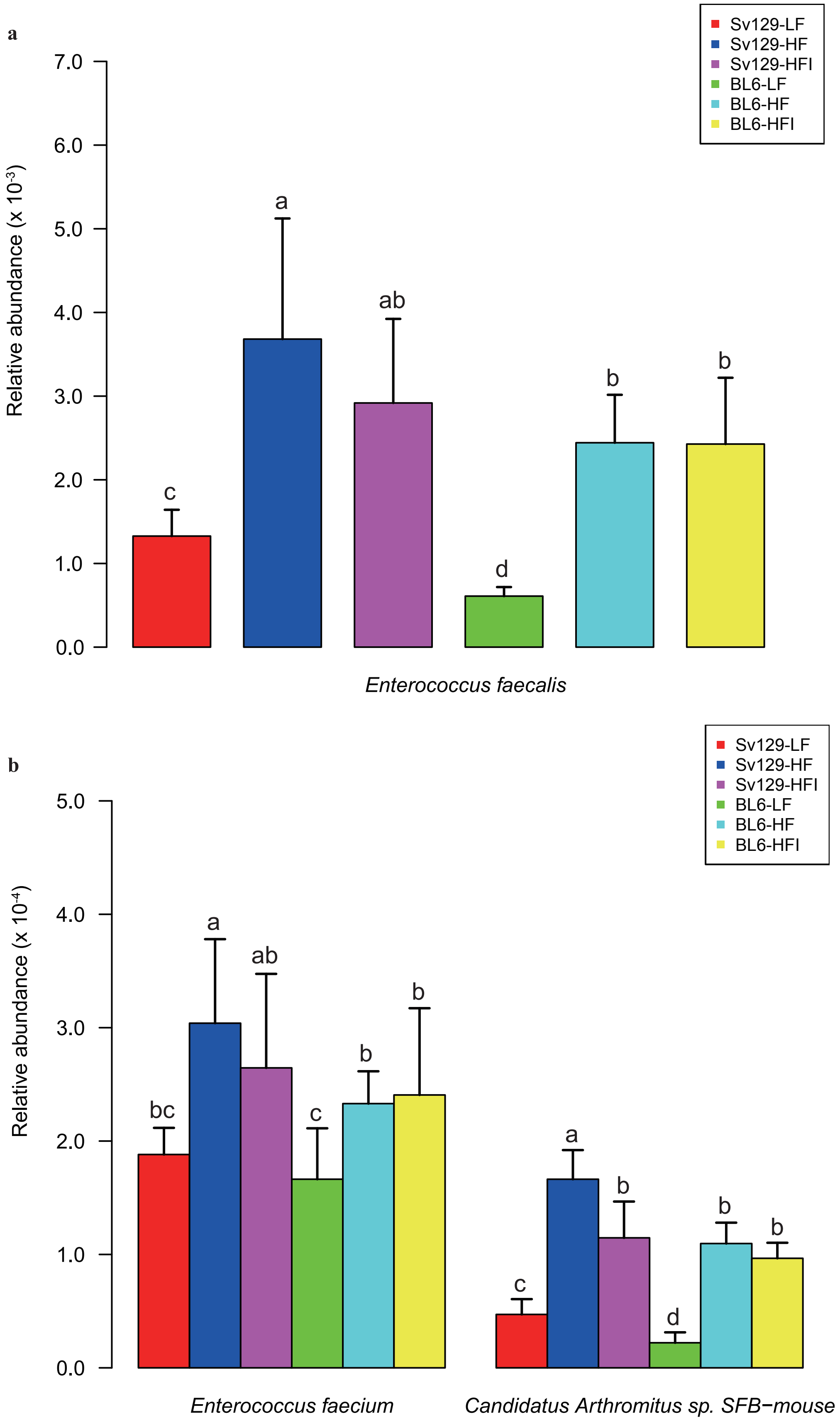

Figure S14. Relative abundance of Enterococcus faecalis-related bacteria and segmented filamentous bacteria. The abundance of Enterococcus faecalis-related bacteria and segmented filamentous bacteria has been reported to be affected by administration of indomethacin. The relative abundance of these bacteria was increased in HF fed mice of both strain, but no effect of indomethacin was observed. Statistical differences were analyzed by unpaired Wilcoxon Rank-Sum test (with FDR correction). Statistically significant differences ($P < 0.05$) between groups are denoted with different letters (a, b, c, d) on the top of the graphic boxes.
